# Rapid metabolic reprogramming mediated by the AMP-activated protein kinase during the lytic cycle of *Toxoplasma gondii*

**DOI:** 10.1038/s41467-023-36084-0

**Published:** 2023-01-26

**Authors:** Yaqiong Li, Zhipeng Niu, Jichao Yang, Xuke Yang, Yukun Chen, Yingying Li, Xiaohan Liang, Jingwen Zhang, Fuqiang Fan, Ping Wu, Chao Peng, Bang Shen

**Affiliations:** 1 State Key Laboratory of Agricultural Microbiology, College of Veterinary Medicine, Huazhong Agricultural University, Wuhan, Hubei Province 430070 PR China; 2 National Facility for Protein Science in Shanghai, Zhangjiang Lab, Shanghai Advanced Research Institute, Chinese Academy of Science, Shanghai, 201210 PR China; 3 Key Laboratory of Preventive Medicine in Hubei Province, Wuhan, Hubei Province 430070 PR China; 4 Hubei Hongshan Laboratory, Wuhan, Hubei Province 430070 PR China

**Keywords:** Parasite biology, Cellular microbiology

## Abstract

The ubiquitous pathogen *Toxoplasma gondii* has a complex lifestyle with different metabolic activities at different stages that are intimately linked to the parasitic environments. Here we identified the eukaryotic regulator of cellular homeostasis AMP-activated protein kinase (AMPK) in *Toxoplasma* and discovered its role in metabolic programming during parasite’s lytic cycle. The catalytic subunit AMPKα is quickly phosphorylated after the release of intracellular parasites to extracellular environments, driving energy-producing catabolism to power parasite motility and invasion into host cells. Once inside host cells, AMPKα phosphorylation is reduced to basal level to promote a balance between energy production and biomass synthesis, allowing robust parasite replication. AMPKγ depletion abolishes AMPKα phosphorylation and suppresses parasite growth, which can be partially rescued by overexpressing wildtype AMPKα but not the phosphorylation mutants. Thus, through the cyclic reprogramming by AMPK, the parasites’ metabolic needs at each stage are satisfied and the lytic cycle progresses robustly.

## Abstract (web summary)

Efficient metabolic regulation is key for parasite growth. Here, the authors report that *Toxoplasma* alters its AMPK phosphorylation during the lytic cycle, which reprograms parasite’s metabolism to ensure metabolic needs at different stages are met.

## Introduction

Changes in environmental conditions are challenges all living organisms must deal with. Due to the unique life style, parasitic organisms are particularly proficient in responding and adapting to environmental changes. *Toxoplasma gondii*, a ubiquitous protozoan infecting one third of the world’s human population and numerous animals, is able to grow and survive in extremely diverse host and environmental conditions^1,2^. This parasite has a complex life cycle alternating between multiple stages that are key for its pathogenesis and transmission. During acute infection of intermediate hosts, the parasites proliferate rapidly as tachyzoites, which are responsible for the clinical symptoms of toxoplasmosis^3^. Tachyzoites actively invade host cells, replicate in them and then lyse them to start new invasions when the parasite number reaches a certain amount. Under optimal conditions, the parasites continuously grow as tachyzoites and repeat the lytic cycle. However, under stress or starvation conditions, tachyzoites may convert to a less active form called bradyzoites, which are enclosed in tissue cysts and maintain a life-long chronic infection in hosts^1,2,4^.

*Toxoplasma* parasites have different metabolic activities at different stages. The majority of glycolytic enzymes have two isoforms, many of which exhibit stage specific expression^5,6^, indicating distinct requirements of glycolytic activity at different stages. Similarly, bradyzoites and oocysts accumulate large amounts of amylopectin, which is barely seen in tachyzoites^7–9^. The physiological significance and underlying regulatory mechanisms for such stage specific metabolism are largely unknown. The lytic cycle of tachyzoites contains two stages, a short extracellular stage when freshly egressed parasites use gliding motility to find and invade host cells, and an intracellular stage when invaded parasites proliferate within host cells. From the metabolic point of view, the primary goal of extracellular tachyzoites is to generate enough energy that can power a fast and efficient invasion, whereas intracellular parasites need balanced energy production and macromolecule synthesis to replicate themselves. The glycolytic enzyme fructose-bisphosphate aldolase has been observed to re-localize from cytoplasm to the parasite periphery as soon as the parasites are released from host cells^10^. As the motor complex that drives parasite motility is underneath parasite’s membrane, the re-localization of glycolytic enzymes was thought to be a way of quickly generating energy in places where it is needed^11^. In addition, treating tachyzoites with ^13^C-labeled glucose to monitor the flux of glucose derived carbon, it has been shown that although ^13^C could be efficiently incorporated into macromolecules like fatty acids in intracellular parasites, it was barely incorporated into such molecules in extracellular parasites^12^. These observations suggest that, although the extracellular stage is very brief lasting seconds to minutes, its metabolism is fundamentally different than that of intracellular parasites. How such short-time metabolic transition is regulated and achieved is completely unknown.

Maintaining metabolic homeostasis under different conditions is key to the life of all organisms. In eukaryotes, the AMP-activated protein kinase (AMPK) is a crucial regulator of cellular metabolism that ensures the metabolic activities in a cell meet its needs^13,14^. AMPK is a conserved serine/threonine kinase consist of three subunits, a catalytic subunit α and two regulatory subunits β and γ. It monitors the energy status in a cell by sensing the AMP to ATP ratio and then turns on or off the energy producing or consuming metabolic programs accordingly^15,16^. AMPKγ contains cystathionine-β-synthase (CBS) domains that bind AMP, therefore enabling the AMPK complex to respond to changes in energy level^17,18^. Binding of AMP to AMPKγ induces the phosphorylation of AMPKα at the T172 residue (residue numbering is based on a rat AMPKα) and activates the kinase activity of AMPKα^18,19^. Regulation of AMPK activity also requires the β subunit (AMPKβ), which functions as a structural core for the AMPK complex by interacting with both the α and γ subunits, via its αγ-subunit-binding sequence (αγ-SBS). In addition, its carbohydrate-binding module (CBM), and post-translational modifications like myristoylation and phosphorylation also play roles to tune the activity of AMPK^20^. Once activated, AMPK initiates a network of signaling events that reprogram cellular metabolism to preserve energy. AMPK may phosphorylate target enzymes like acetyl-CoA carboxylase to modulate their activities^13,14,19^. Transcriptional regulation of target gene expression is another way for AMPK to regulate metabolism and cell growth^21^. Hundreds of targets have been identified for AMPK from yeasts to humans^14,16,22^. Depending on the signals, AMPK fine tunes the activities of multiple targets to achieve a concerted metabolic program in response to environmental fluctuations.

The regulatory roles of AMPK on metabolism and cellular homeostasis are extensively studied in model eukaryotes like *Arabidopsis*, yeasts, and mice. Yet, its functions in parasitic organisms are rarely investigated. AMPK complexes have been identified in *Trypanosoma brucei* and *Trypanosoma cruzi*^23,24^. In *T. brucei*, AMPKα1 is activated during the differentiation from the proliferative form in mammals to the quiescent form pre-adapted for the insect vector. Artificial stimulation or inhibition of AMPKα1 activation significantly affect the transition process, suggesting a role of AMPK in regulating the life styles in this parasite^23^.

In this study, we describe the AMPK complex in *T. gondii* and report its essential function for parasite proliferation. *Toxoplasma* encodes a single canonical AMPK complex that consists of three subunits. Phosphorylation of *Toxoplasma* AMPKα is low in intracellular parasites but it is greatly induced in extracellular parasites, leading to cyclic activation and deactivation of AMPK during the lytic cycle. By generation and characterization of an AMPKγ depletion mutant that has impaired AMPKα phosphorylation, our results reveal important metabolic reprogramming roles of AMPK during the lytic cycle of tachyzoites.

## Results

### The AMPK complex in *Toxoplasma*

**Fig. 1:**
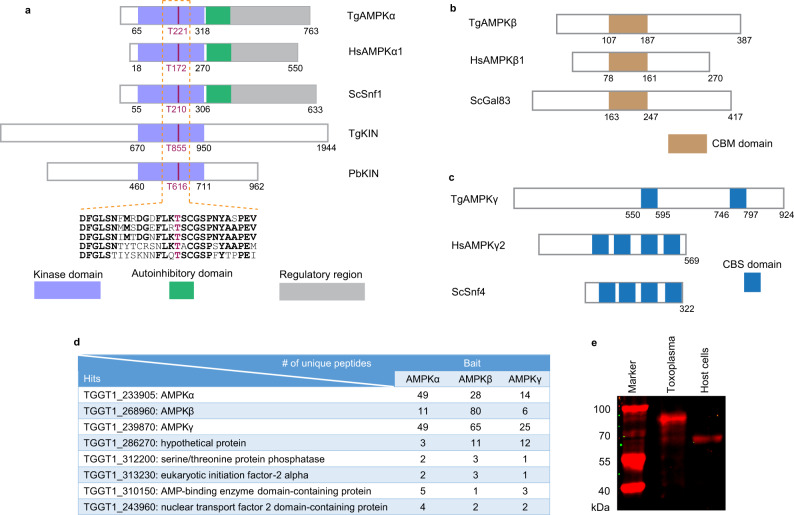
Identification and characterization of the domain structures of the AMPK complex in *T. gondii*. **a–c** domain structures of the three subunits of *Toxoplama* AMPK compared with their corresponding orthologs in other eukaryotes. The human (*Homo sapiens*, Hs), yeast (*Saccharomyces cerevisiae*, Sc) and *Plasmodium* (*Pasmodium berghei*, Pb) proteins are included for comparison. A sequence alignment (done by Clustal X2) of the activation loop in the kinase domain of AMPKα, along with the conserved threonine residue that is subjected to phosphorylation regulation is provided in **a**. **d** transgenic strains expressing TgAMPKα-Ty, TgAMPKβ-Ty or TgAMPKγ-HA were individually used in co-IP (using antibodies against the corresponding epitope tags) experiments to identify proteins interacting with each of the *Toxoplasma* AMPK subunits. Selected hits from these co-IP experiments identified by mass spectrometry (MS) were shown and the numbers denote the number of unique peptides derived from MS analyses that matched the corresponding hits. All these hits were not identified in the control experiments involving untagged parental strains and a full list of the hits are provided in Supplementary data 1. **e** recognition of TgAMPKα by the phospho-AMPKα(Thr172) monoclonal antibody that recognizes phosphorylated HsAMPKα, as determined by Western blotting on purified extracellular parasites (Toxoplasma) and host cells. Source data are provided as a Source data file.

The canonical AMPK complex is a heterotrimer consisting of an α subunit that is an active kinase, and two regulatory subunits β and γ that regulate the activity of α^22^. To identify the AMPK complex in *T. gondii*, AMPK subunits from yeast and human were used as baits to BLASTP search the *Toxoplasma* genome database (TGGT1 of ToxoDB Release 42). Two proteins with significant homologies to human and yeast AMPKα were identified and they were named TgAMPKα (TGGT1_233905) and TgKIN (TGGT1_291050) respectively, which are candidates of AMPKα orthologues in *Toxoplasma*. Further sequence analyses indicate that TgAMPKα has classic domain structures as other AMPKα proteins. It has a conserved kinase domain at the N terminus, followed by an autoinhibitory domain and regulatory motifs at the C terminal region (Fig. 1a). TgKIN also contains a kinase domain. However, it has long extensions in the N and C terminal regions that are poorly conserved (Fig. 1a), similar to the KIN kinases in *Plasmodium* species that do not seem to have AMPKβ or AMPKγ. Judging from the domain structure, TgAMPKα is likely the bona fide AMPKα orthologue in *Toxoplasma*, whereas TgKIN is orthologous to *Plasmodium* KIN and may function independently of the AMPK subunits (further evidence supporting this is described below). BLAST searches of ToxoDB also identified proteins that are likely orthologous to human and yeast AMPKβ (TGGT1_268960, TgAMPKβ) and AMPKγ (TGGT1_239870, TgAMPKγ), but in both cases the homology is confined to short regions (Fig. 1b, c). The putative TgAMPKβ contains a CBM domain, which showed 46% sequence identity with that of human AMPKβ1 (Fig. 1b). Similarly, the putative TgAMPKγ contains two CBS domains that are commonly found in AMPKγ and they had 21.7% and 15.2% sequence identity with the first CBS domain of human AMPKγ2 respectively (Fig. 1c).

To further explore the AMPK complex in *Toxoplasma*, a Ty tagged transgenic strain RH *Δhxgprt*/LoxP-AMPKα-Ty, in which the endogenous AMPKα was replaced with a Ty tagged AMPKα expressed from a pTub promoter, was constructed (Fig. S1a–c). This strain was then used in a co-immunoprecipitation (co-IP, using the Ty monoclonal antibody) and mass spectrometry (MS) experiment to search for proteins interacting with AMPKα. Judging from the number of unique peptides from the MS results, the putative TgAMPKγ identified from the bioinformatic analysis was the top 1 hit from this AMPKα-Ty based co-IP/MS (Fig. 1d and table S1). No peptides from TgAMPKγ were found in the control experiment using the untagged parental strain RH. Similarly, the putative AMPKβ was also among the top 10 hits (Fig. 1d and Table S1). To further confirm these proteins do form a complex, the putative AMPKβ and AMPKγ subunits were also subjected to co-IP/MS analyses. The RH *Δhxgprt*/LoxP-AMPKβ-Ty (which contained a Ty tagged AMPKβ, Fig. S2) and the AMPKγ-mAID (which contained an HA tagged AMPKγ, described below) strains were used for this purpose in the co-IP experiments, using anti-Ty and -HA antibodies respectively. When the MS results from these co-IP experiments were analyzed, the putative TgAMPK subunits always came up as top hits (Fig. 1d and table S1), suggesting that they are bona fide AMPK subunits that form the AMPK complex in *T. gondii*. In addition, these co-IP experiments also identified potential binding partners of the *Toxoplasma* AMPK complex. Particularly, the 63 proteins that were enriched in all three AMPK subunits-based co-IPs are likely to interact with AMPK (Fig. S3, Table S1 and Supplementary data 1). On the other hand, none of these co-IPs identified TgKIN as a hit, implying that TgKIN likely functions by itself and does not interact with any of the putative AMPK subunits. Taken together, our data suggest that *Toxoplasma* tachyzoites have one AMPK complex consisting of TgAMPKα, TgAMPKβ and TgAMPKγ.

The activity of the AMPK complex is regulated by phosphorylation of a specific threonine residue (T172 for mammalian AMPK) in the activation loop of AMPKα. Because of the high sequence similarity in this region (Fig. 1a), a monoclonal antibody that reacts with phosphorylated human AMPKα at threonine 172 also recognized phosphorylated TgAMPKα in purified extracellular parasites (Fig. 1e). To further confirm the parasite product recognized by this antibody is truly TgAMPKα, we fused a mini auxin-inducible degron (mAID) to the carboxyl terminus of endogenous TgAMPKα and constructed an AMPKα-mAID strain, whose AMPKα could be depleted after indole-3-acetic acid (IAA) treatment (Fig. S4a). As expected, the product recognized by the anti-phospho-AMPKα(Thr172) antibody disappeared after the AMPKα-mAID parasites were treated with IAA (Fig. S4b), demonstrating that the parasite product recognized is indeed TgAMPKα.

### AMPKγ is essential for parasite growth

**Fig. 2:**
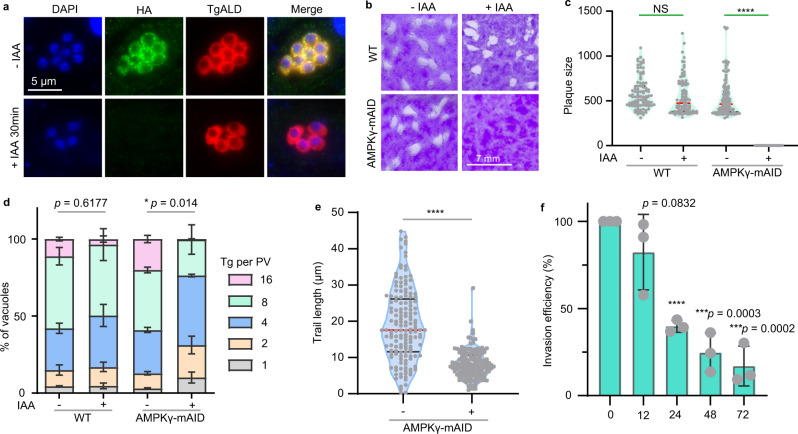
AMPKγ is essential for the growth of *Toxoplama* tachyzoites. **a** Depletion of TgAMPKγ in the AMPKγ-mAID strain by IAA treatment, as determined by IFA on intracellular parasites treated with or without IAA. The anti-HA antibody was used to probe the level of AMPKγ and TgALD was used as cytoplasmic marker. **b** Lack of parasite growth after TgAMPKγ depletion, as determined by plaque assays with or without IAA treatment for 7 days. **c** Relative sizes of plaques (expressed as pixel units when calculated from Adobe Photoshop) derived from **b**. More than 90 plaques were determined for each condition and the data are graphed as violin plots (median with interquartile range). ****P < 0.0001, NS (*p* = 0.1604): not significant, unpaired two-tailed Student’s *t*-test. **d** Intracellular replication rates of indicated strains with or without IAA treatment, as determined by the distribution of parasitophorous vacuoles (PVs) containing 1, 2, 4, 8 or 16 parasites after 24 h of intracellular growth. Means ± SEM of *n* = 3 independent experiments, each with two technical replicates, two-way ANOVA with Tukey’s multiple comparisons post-tests. **e** The length of trails formed by extracellular parasites gliding on bovine albumin coated surface. The parasites were pretreated with or without IAA for 48 h, mechanically released from host cells and used to glide on cover slips precoated with bovine albumin. The trails formed by parasite gliding was determined by IFA using an antibody against the surface protein SAG1. Over 150 gliding trails from *n* = 3 independent experiments (each contained 49 to 67 trails) were analyzed for each condition and graphed as violin plots (median with interquartile range). ***P < 0.001, unpaired two-tailed Student’s *t*-test. **f** Invasion efficiencies of the AMPKγ-mAID strain pretreated with IAA for 0, 12, 24, 48 or 72 h during the intracellular growth stage. Pretreated parasites were purified and used to invade HFF cells for 20 min and then a two-color staining assay was used to distinguish invaded parasites from non-invaded ones. Invasion efficiency of non-treated parasites (0 h) was set as 100% and used to normalize that of IAA treated parasites. Means ± SD of *n* = 3 independent experiments, ****P < 0.0001, unpaired two-tailed Student’s *t*-test, each was compared with the 0 h group. Source data are provided as a Source data file.

To assess the physiological roles of AMPK in *Toxoplasma*, we first introduced a plasmid expressing the Cre recombinase into the RH *Δhxgprt*/LoxP-AMPKα-Ty strain described above. Cre recombinase induced excision of AMPKα and activated the expression of YFP, which reported AMPKα deletion (Fig. S1c). We have tried to obtain a clean YFP^+^ AMPKα^-^ clone after transfection of the Cre expressing plasmid but failed, implying that AMPKα may be essential for parasite growth. Further supporting this notion, the YFP^+^ population in the pool (RH *Δhxgprt*/LoxP-AMPKα-TY transfected with Cre) peaked 2 days after Cre introduction and then gradually declined and dropped to nearly zero at day 8 (Fig. S1d), indicating that AMPKα^-^ mutants had severe growth defects so that they could not compete with the AMPKα^+^ parasites in the pool. Nonetheless, it is difficult to use this system to investigate the role of TgAMPKα, since the percentage of YFP^+^ AMPKα^-^ parasites was very low (<10% even at the peak point) after Cre transfection (Fig. S1d), making it difficult to obtain enough AMPKα^-^ parasites for downstream work. Meanwhile, the above mentioned AMPKα-mAID strain was also not ideal for functional assessment of AMPKα, because IAA treatment did reduce parasite growth but did not arrest it as the YFP^+^ AMPKα^-^ parasites derived from the loxP-AMPKα/Cre system did. This is likely due to the incomplete depletion of AMPKα by IAA. Alternatively, we decided to focus on the γ subunit and used genetic approaches to dissect its biological functions. In other model organisms, AMPKγ senses changes in cellular metabolism and is essential to regulate the kinase activity of AMPKα^25,26^. As such, AMPKγ is key for the activity of the AMPK complex and we focused on AMPKγ to assess the role of AMPK in *Toxoplasma* parasites. We first targeted *AMPKγ* for direct gene deletion, using CRISPR/Cas9 assisted homologous gene replacement. However, knockout of this gene failed despite having tried multiple times, implying a critical role of AMPKγ for parasite growth. Then we switched to a conditional depletion approach by adding an mAID to the carboxyl terminus of AMPKγ at the endogenous gene locus in the Tir1 expressing strain RH *∆hxgprt* Tir1^27^ (Fig. S5a). The resulted AMPKγ-mAID strain was confirmed by diagnostic PCRs (Fig. S5b) and addition of IAA to culture medium induced efficient degradation of AMPKγ, as detected by immunofluorescent assays (Fig. 2a) and Western blotting (Fig. S5c). AMPKγ depletion suppressed parasite growth, as demonstrated by the lack of plaque formation of the IAA treated AMPKγ-mAID parasites in HFF monolayers (Fig. 2b, c). Detailed analyses suggested that AMPKγ depletion had global effect on the lytic cycle of parasites. Efficiencies of intracellular replication (Fig. 2d), gliding motility (Fig. 2e), and host cell invasion (Fig. 2f) were all reduced after AMPKγ degradation. The impact of AMPKγ depletion on these parasite activities is probably indirect, because AMPKγ degradation could be induced by IAA within 30 min (Fig. 2a), yet the invasion defects did not occur until 24 h after IAA treatment (Fig. 2f).

To further confirm the growth defects of the IAA treated AMPKγ-mAID mutant is caused by the absence of AMPKγ, a complementation strain (Comp-γ) expressing a Ty tagged AMPKγ from the *UPRT* locus of the AMPKγ-mAID strain was constructed (Fig. S6a). Diagnostic PCRs confirmed the correct integration of the complementation construct. IFA and Western blotting both assured the expression of the complementing AMPKγ (Fig. S6b–d). In contrast to the AMPKγ-mAID strain that did not grow in the presence of IAA, AMPKγ complementation fully restored parasite growth and IAA treatment did not affect plaque formation of the Comp-γ strain (Fig. S6e), suggesting that AMPKγ deficiency is indeed responsible for the growth arrest of the IAA treated AMPKγ-mAID mutant.

### AMPKγ repression abolishes AMPKα phosphorylation

**Fig. 3:**
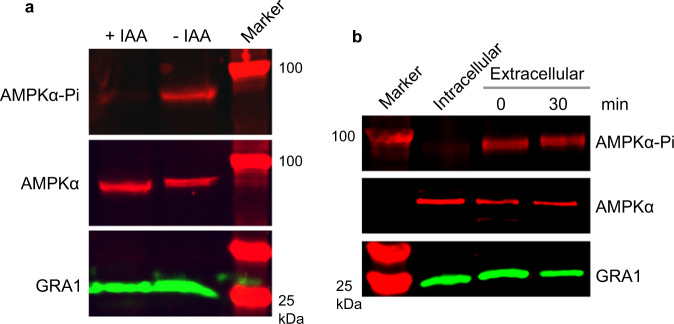
Assessment of TgAMPKα phosphorylation under different conditions. **a** TgAMPKα phosphorylation is abolished in TgAMPKγ depleted parasites. The AMPKγ-mAID parasites pretreated with or without IAA for 48 h were forced to egress from host cells and then analyzed by Western blots, using antibodies that recognized phosphorylated AMPKα, total TgAMPKα or TgGRA1. **b** TgAMPKα phosphorylation is induced in extracellular parasites in the wildtype strain RH. Intracellular parasites (intact PV contained in host cells) or mechanically released parasites incubated in extracellular environment for 0 or 30 min were examined by Western blotting. Source data are provided as a Source data file.

Phosphorylation of the T172 residue (or equivalent) is key to regulate the activity of canonical AMPK complexes. Since TgAMPKγ depletion is lethal in parasites, we sought to determine its impact on AMPKα phosphorylation. When mechanically released extracellular parasites were examined by Western blotting using the phospho-AMPKα(Thr172) antibody, phosphorylation of TgAMPKα at the T221 residue (corresponding to mammalian T172) was found to be abolished after AMPKγ depletion, although the protein level of TgAMPKα was not affected (Fig. 3a). The reduction of TgAMPKα phosphorylation was fully restored by AMPKγ complementation (Fig. S7), confirming that TgAMPKγ is essential for the phosphorylation of TgAMPKα.

To screen for conditions that change the phosphorylation status of TgAMPKα, it was found that release of intracellular parasites into extracellular environments induced TgAMPKα phosphorylation. When intracellular parasites (samples contained host cells with intact parasitophorous vacuoles) were subjected to Western blot analysis using the phospho-AMPKα(Thr172) antibody, TgAMPKα phosphorylation could be barely detected. In contrast, when infected host cells were passed through a 22-gauge needle to lyse the host cells and release the parasites to culture medium, TgAMPKα phosphorylation was quickly and dramatically induced (Fig. 3b). Incubating the mechanically released parasites in the extracellular environment for longer time did not obviously increase TgAMPKα phosphorylation further. Together, these results suggest that release of parasites from host cells into extracellular environments is a strong signal to activate TgAMPKα phosphorylation.

### AMPKα partially rescues the growth defects of the AMPKγ depletion mutant

**Fig. 4:**
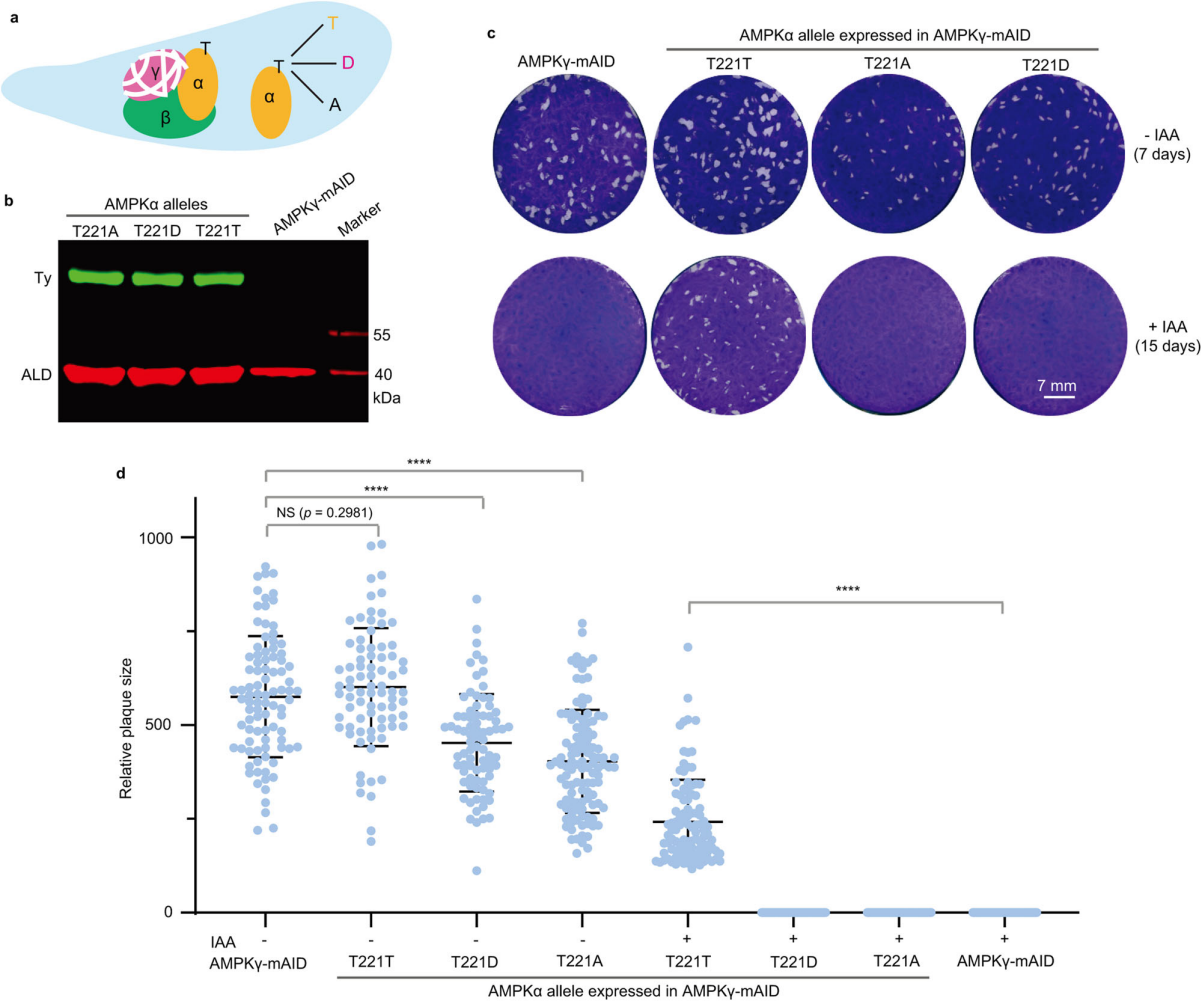
Impact of the expression of different alleles of TgAMPKα on the growth of TgAMPKγ depleted parasites. **a** Schematic illustration of expressing the TgAMPKα-T221T, -T221D, or -T221A alleles in parasites lacking TgAMPKγ, which was achieved by IAA treatment of the AMPKγ-mAID mutant. The AMPKα expressing cassette was inserted into the *UPRT* locus of the AMPKγ-mAID strain to achieve similar expressions for different AMPKα alleles. **b** Expression levels of the TgAMPKα-T221T/D/A alleles in AMPKγ-mAID, as determined by Western blotting against the Ty tag fused to TgAMPKα. TgALD was included as a loading control. **c** Plaque assays assessing the overall growth of the AMPKγ-mAID mutant expressing different alleles of TgAMPKα, with or without IAA treatment for 7 or 15 days. **d** Sizes of plaques derived from **c**, expressed as pixel units when calculated by Adobe Photoshop. Means ± SD of *n* ≥ 79 plaques for each condition. ****P < 0.0001, NS: not significant, unpaired two-tailed Student’s *t*-test. Source data are provided as a Source data file.

Decreased phosphorylation of TgAMPKα upon AMPKγ depletion prompted us to check whether the change of TgAMPKα phosphorylation was responsible for the growth defects of the AMPKγ depleted mutant. To this end, we introduced three different alleles of TgAMPKα that mimic different phosphorylation states into the AMPKγ-mAID mutant (Fig. 4a). The T221D allele mimics phosphorylation, whereas T221A is phosphorylation defective. The wildtype T221T allele was included as a control. These different alleles of TgAMPKα were individually inserted into the *UPRT* locus of the AMPKγ-mAID strain and expressed at similar levels (Fig. 4b). The impact of these AMPKα alleles on the growth of the AMPKγ conditional depletion strain was estimated by plaque assays. Without IAA treatment, expression of TgAMPKα-T221D and TgAMPKα-T221A in AMPKγ-mAID modestly, but significantly reduced parasite growth, as indicated by smaller plaques (Fig. 4c, d). On the other hand, expression of a second copy of TgAMPKα (the T221T allele) did not have an obvious impact (Fig. 4c, d). When IAA was used to induce AMPKγ degradation, neither TgAMPKα-T221D nor TgAMPKα-T221A improved the growth of the AMPKγ depleted mutants, as no visible plaques were detected even after 15 days growth. On the other hand, expression of TgAMPKα-T221T (wildtype TgAMPKα) allowed plaque formation in the presence of IAA (Fig. 4c, d), suggesting robust rescue of AMPKγ depleted mutants by TgAMPKα.

### Change of the AMPKα phosphorylation status affects the parasites’ metabolic adaption during the lytic cycle

**Fig. 5:**
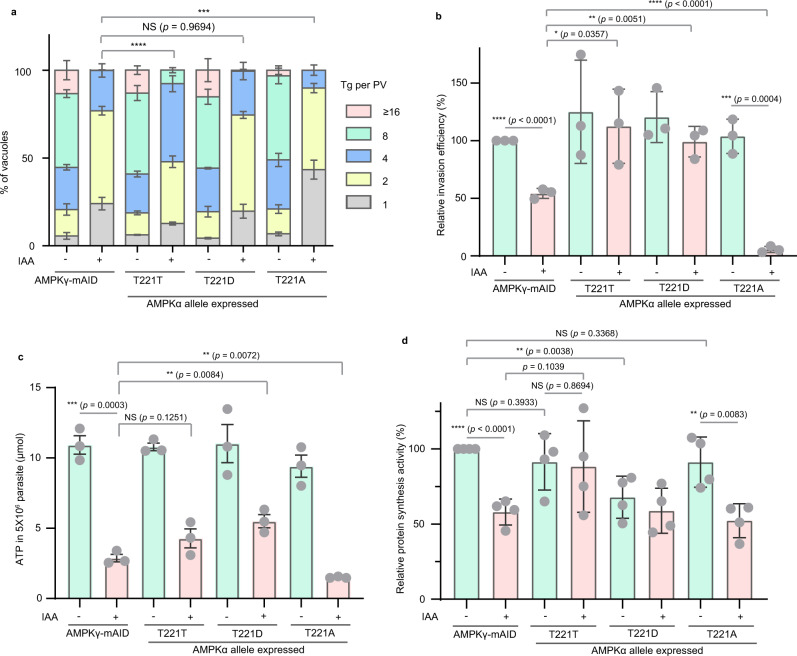
Effects of the expression of different alleles of TgAMPKα on the intracellular replication (a), invasion efficiency (b), ATP level (c), and nascent protein synthesis activity (d) of TgAMPKγ depleted parasites. All experiments were repeated *n* = 3 (**a**–**c**) or *n* = 4 (**d**) times independently and the Means ± SEM are graphed. ***P = 0.0004, ****P < 0.0001, two-way ANOVA with Tukey’s multiple comparisons post-tests (**a**), unpaired two-tailed Student’s *t*-test (**b**–**d**). The replication and invasion assays were performed as in Fig. 2d and f. The ATP level was measured by a commercial luciferase assay using lysates prepared from 5 × 10^6^ mechanically released fresh parasites that were treated with or without IAA for 48 h. The nascent protein synthesis activity was determined by adding L-homopropargylglycine, an alkyne-tagged analog of methionine, to parasites that were treated with or without IAA. Then the incorporated L-homopropargylglycine was detected by a click chemistry approach using the fluorescent probe FAM azide and quantified by flow cytometry analyses. Source data are provided as a Source data file.

The different capacities of TgAMPKα alleles to rescue the growth of AMPKγ depleted mutants allowed us to examine how AMPKα phosphorylation affects parasite activities. The above-described plaque assays assess the overall fitness of the parasites. Defects in any step of the lytic cycle, such as invasion and replication, would lead to changes in plaque formation. To examine the impact of AMPKα phosphorylation on each of these steps, we first checked the efficiency of intracellular replication by determining the number of parasites in the PVs. Without IAA treatment, expression of T221T or T221D of TgAMPKα had little effect on parasite proliferation, whereas T221A slightly reduced proliferation (Fig. 5a). When treated with IAA to deplete AMPKγ, expression of TgAMPKα-T221T significantly increased the rates of parasite replication (compared with AMPKγ-mAID treated with IAA), whereas T221D or T221A did not (Fig. 5a). Expression of the T221A allele actually reduced the replication of AMPKγ depleted mutants (Fig. 5a). On the other hand, when the efficiency to invade host cells was tested by a 20-min invasion assay, both the T221T and T221D alleles were able to fully restore the invasion defects of AMPKγ depleted mutants. The T221A allele, in contrast, further reduced invasion efficiency instead of improving it (Fig. 5b), implying a dominant negative effect of this allele. Together, these results demonstrated that the T221T allele of TgAMPKα restored host cell invasion and improved the rate of intracellular replication. Therefore, it was able to partially rescue the growth of AMPKγ depleted mutants. The T221D allele restored invasion but not replication, whereas T221A did neither, explaining their incapability to rescue growth.

One key function of the AMPK complex is to regulate metabolic homeostasis. To check the impact of AMPKγ depletion and subsequent expression of TgAMPKα alleles on the energy status of parasites, cellular ATP levels were estimated using a firefly luciferase-based ATP bioluminescence assay kit. Treating the AMPKγ-mAID parasites with IAA for 48 h reduced ATP level by 73.8% ± 1.8% (Fig. 5c). In addition, the decrease of cellular ATP was exacerbated as the time of IAA treatment increased (Fig. S8), suggesting that the drain of ATP after AMPKγ depletion is time accumulative. Expression of the wildtype or the mutant forms of TgAMPKα did not significantly alter ATP levels in the absence of IAA. With IAA treatment, the T221D allele of TgAMPKα significantly restored cellular ATP level, although not to the level in wildtype parasites (Fig. 5c). Expression of TgAMPKα-T221T also increased the ATP level in TgAMPKγ depletion mutants, but the increase is not statistically significant due to the high variation between experiments during ATP quantification (Fig. 5c). In contrast, expression of the T221A allele further reduced the ATP level, consistent with a dominant negative effect of this mutant (Fig. 5c). Interestingly, the effect of different alleles of TgAMPKα on cellular ATP levels matches well with that on the invasion efficiency of corresponding strains. While AMPK is well known to regulate catabolic and anabolic activities to achieve energy homeostasis, we also assessed the macromolecule synthesis activity of the TgAMPKγ depletion mutants, using nascent protein synthesis as an example. A click-chemistry approach detecting the incorporation of an alkyne-tagged analog of methionine (L-homopropargylglycine) into nascently synthesized proteins was used for this purpose. AMPKγ depletion by IAA treatment of the AMPKγ-mAID strain reduced nascent protein synthesis by 42.0% ± 8.7% (Fig. 5d). Interestingly, expression of AMPKα-T221D resulted in lower protein synthesis activity even without IAA treatment, whereas AMPKα-T221A or AMPKα-T221T did not have such effect (Fig. 5d). In the presence of IAA, expression of AMPKα-T221D or AMPKα-T221A in the AMPKγ-mAID strain did not improve its protein synthesis rates (Fig. 5d). On the other hand, expression of AMPKα-T221T increased the protein synthesis activity to the level that was similar in AMPKγ-mAID before IAA treatment (Fig. 5d), although this increase was not typically treated as statistically significant with a *p*-value of 0.1039. The different effects of AMPKα alleles on the ATP production and protein synthesis activities of AMPKγ depleted parasites suggest that the phosphorylation status of AMPKα had a significant impact on parasite metabolism.

### Chemical activation or inhibition of AMPK have distinct impacts on parasite replication and invasion

**Fig. 6:**
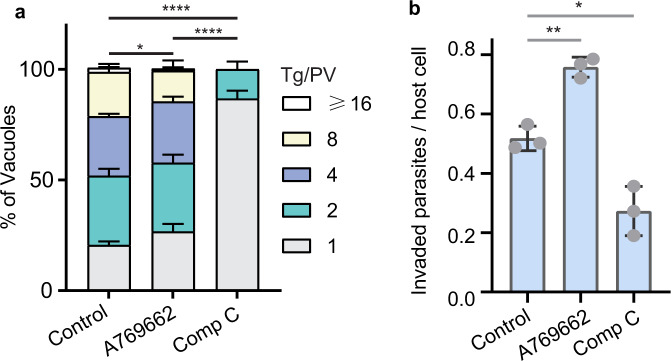
Effects of the AMPK activator A769662 and inhibitor compound C (Comp C) on parasite replication and invasion. **a** intracellular replication assays of RH in the presence of indicated treatments, as done in Fig. 2d. Invaded parasites were allowed to replicate for 24 h in MEF cells lacking both AMPKα1 and AMPKα2. Means ± SEM of *n* = 3 independent experiments, ****P < 0.0001, *P = 0.0201, two-way ANOVA with Tukey’s multiple comparisons post-tests. **b** efficiency of host cell invasion determined by a two-color assay that discriminated invaded vs non-invaded parasites. The RH parasites were treated with indicated chemicals for 1 h before egress. Then they were released by needle passage and purified parasites were used to infect HFF cells for 10 min in the presence of corresponding compounds. Subsequently the number of invaded parasites were determined by IFA. Invasion efficiency was plotted as the number of invaded parasites divided by the number of host cells. Means ± SD of *n* = 3 independent experiments, *P = 0.0103, **P = 0.0015, unpaired two-tailed Student’s *t*-test. Source data are provided as a Source data file.

Above results from genetic studies demonstrated the key roles of TgAMPKα phosphorylation in regulating parasite activities and metabolism during the lytic cycle. To further examine the role of AMPK, we used a chemical genetic approach that involved the use of two widely used compounds to activate and inhibit AMPK respectively. A769662 was used as an activator because of its potent and direct activation of mammalian AMPK, whereas Compound C (Dorsomorphin dihydrochloride) was used as an inhibitor. To rule out the possibility that these compounds targeted host AMPK to affect parasite activities, we used a mouse embryonic fibroblast (MEF) cell line whose AMPKα1 and AMPKα2 were both disrupted^28^. Treating intracellular *Toxoplasma* parasites with A769662 or Compound C reduced their replication in MEF cells lacking AMPKα (Fig. 6a). The inhibitory effect of Compound C was significantly stronger than that of A769662 and the treated parasites barely replicated (Fig. 6a). On the other hand, when the invasion efficiency of extracellular parasites was compared, treating parasites with A769662 for 1 h increased the efficiency of host cell invasion by 46.8% ± 10.3%. In contrast, Compound C treatment decreased the invasion efficiency to 53.7% ± 19.8% of that receiving no treatments (Fig. 6b). These results are consistent with the above data from genetic studies, further supporting that AMPK activation boosts invasion of extracellular parasites and lytic cycle progression requires dynamic AMPK activation.

### AMPKγ depletion alters the phosphorylation of proteins involved in diverse metabolic pathways

Transcriptional regulation and post-translational modification of targets are two common strategies applied by AMPK to regulate cellular activities. In order to understand the molecular mechanisms underlying the poor growth and impaired metabolism of the AMPKγ depleted mutants, we first checked the parasites’ gene expression changes upon AMPKγ depletion. RNA-seq analyses demonstrated that AMPKγ depletion had minor effect on parasite gene expression. Only 61 genes satisfied our criteria of differentially expressed genes calling, among which 40 genes were upregulated and 21 were downregulated in the AMPKγ depletion mutant (Fig. S9, Supplementary Data 2).

**Fig. 7:**
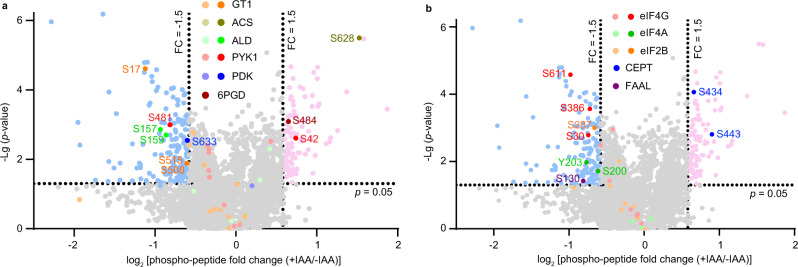
TgAMPKγ depletion affects the phosphorylation of multiple proteins in *Toxoplasma*. Alteration of the phosphorylation of selected sugar catabolism proteins (**a**) and biomass synthesis proteins (**b**) at indicated positions after TgAMPKγ degradation, as determined by phosphoproteomics on needle released AMPKγ-mAID parasites pretreated with or without IAA for 48 h. Extracted parasite proteins from *n* = 3 sets of independent samples (each set contained one sample for IAA treated parasites and one for nontreated parasites) were trypsin digested and labeled with six plex tandem mass tags (TMT). Then, phospho-peptides were enriched by TiO_2_ and IMAC, and quantified by LC-MS/MS. The abundance change (expressed as log_2_ fold change) and the corresponding *p*-value (determined by Empirical Bayes two-tailed tests in the limma package) (expressed as -Lg *p*-value) of each phospho-peptide after TgAMPKγ depletion (+IAA/-IAA) were plotted. Each condition was tested *n* = 3 times independently. GT1 glucose transporter 1, ACS Acetyl-coenzyme A synthetase, ALD fructose-1,6-bisphosphate aldolase, PYK1 pyruvate kinase 1, PDK pyruvate dehydrogenase kinase, 6PGD 6-phosphogluconate dehydrogenase, eIF eukaryotic initiation factor, CEPT choline/ethanolamine phosphotransferase, FAAL Fatty acyl-AMP ligase.

AMPKγ depletion leads to dramatic reduction of AMPKα phosphorylation, which is key to the kinase activity of AMPK. As such, we sought to determine the alteration of protein phosphorylation in parasites upon AMPKγ depletion. Extracellular parasites of the AMPKγ-mAID strain pretreated with or without IAA for 48 h were subjected to proteomic and phospho-proteomic analyses respectively, to assess the changes in protein abundance and phosphorylation status of individual proteins. Quantitative proteomics identified 47 proteins whose abundance was altered >1.5 fold (*p* < 0.05), 24 of which were decreased and 23 were increased after AMPKγ depletion (Fig. S10, Supplementary data 3). On the other hand, the abundance of 325 phospho-peptides had an abundance change over 1.5 fold after AMPKγ depletion. After adjustments with the protein level changes (to rule out the change of phospho-peptides abundance was caused by changes in protein level), the abundance of 285 phospho-peptides corresponding to 170 proteins was changed >1.5 fold (*p* < 0.05) upon AMPKγ depletion (Supplementary data 4). More than 40% (74/170 = 43.5%) of these proteins are hypothetical proteins with unknown functions. Besides those, a large number of proteins with metabolic roles, including enzymes, transporters and regulators, were differentially phosphorylated in the AMPKγ^+^ versus AMPKγ^-^ parasites. Notably, the glycolytic enzymes pyruvate kinase 1 (PYK1) and fructose-1,6-bisphosphate aldolase (ALD), as well as the major glucose importer glucose transporter 1 (GT1) all contained two or more phospho-peptides that had abundance difference between AMPKγ expressing and depleted parasites (Fig. 7a). Other proteins involved in sugar breakdown or energy metabolism, including 6-phosphogluconate dehydrogenase (6PGD), acetyl-coenzyme A synthetase (ACS) and pyruvate dehydrogenase kinase also showed changes in phosphorylation at specific serine residues upon AMPKγ degradation (Fig. 7a, Supplementary data 4). Similarly, a number of proteins and enzymes involved in anabolism such as lipid and protein synthesis also displayed phosphorylation changes after AMPKγ depletion (Fig. 7b, Supplementary data 4). Particularly, the phosphorylation of three eukaryotic initiation factors (eIF2B, eIF4A and eIF4G) that control the initiation of protein translation was reduced upon AMPKγ degradation (Fig. 7b), which is consistent with the impaired nascent protein synthesis of AMPKγ depleted parasites (Fig. 5d).

## Discussion

**Fig. 8:**
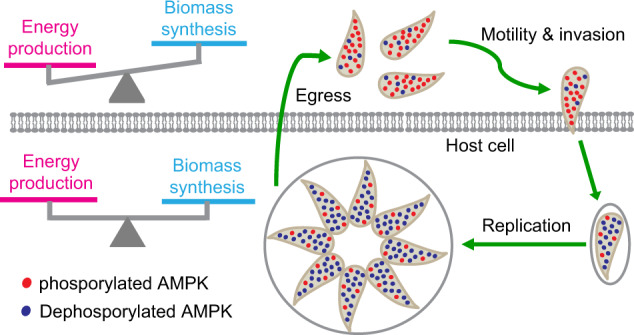
A model for the role of AMPK in reprogramming parasites’ metabolic activities during the lytic cycle. Release of intracellular parasites into extracellular environment induces AMPKα phosphorylation, which activates catabolism to generate energy and suppresses anabolism. At the extracellular stage, the activity of biomass synthesis is low but the parasites need ample energy to power motility for efficient invasion into host cells. Once internalized in host cells, phosphorylation of AMPKα is reduced and a balance between energy producing catabolism and biomass synthesizing anabolism is achieved, enabling robust proliferation of the parasites.

AMPK as an energy sensor and key regulator of metabolism is widely present in almost all eukaryotes and it has been extensively studied in model organisms like yeasts and human cells^13,16^. Nonetheless, little is known about its functions in parasitic organisms, which have unique life styles that require elaborate regulation. Here we have identified an AMPK complex in the obligate intracellular parasite *Toxoplasma gondii*, which is a model to study the complex biology of apicomplexan parasites. Focusing on the γ subunit of the *Toxoplasma* AMPK complex, we reported that an intact AMPK is essential for the growth of this parasite. Conditional depletion of TgAMPKγ resulted in reduced parasite motility, invasion, and intracellular replication (Fig. 2). More importantly, it was found that phosphorylation of TgAMPKα at the T221 residue (therefore activation of the AMPK complex) was greatly induced when the parasites were egressed from the host cells and released into extracellular environment. TgAMPKγ depletion abolished TgAMPKα phosphorylation (Fig. 3). By over-expressing TgAMPKα alleles that mimicked different phosphorylation states in the TgAMPKγ depletion mutant, our results suggest a critical role of AMPK in reprogramming parasite’s metabolic activities during the lytic cycle of *Toxoplasma*. When tachyzoites are egressed from host cells, TgAMPKα is phosphorylated and activated to boost catabolism and suppress anabolism, enabling the parasites to maximize ATP production for efficient motility and invasion. Because at the extracellular stage, biomass synthesis is limited and the most important task is to find suitable host cells and invade them, which requires plenty of energy. After successful invasion and when the parasites are intracellular, the primary goal is to replicate themselves. As such, TgAMPKα phosphorylation is reduced to basal level and the energy production skewed metabolism in extracellular parasites is switched to a more balanced metabolism of energy generation and biomass synthesis, allowing the parasites to proliferate efficiently (Fig. 8). Consistent with this model, while both AMPK inhibitor and activator reduced parasite replication, their impacts on invasion were different. The activator increased the efficiency of invasion, whereas the inhibitor decreased it (Fig. 6).

Our results suggest that there is only one AMPK complex in *Toxoplasma* and each subunit is encoded by one gene. This is different from other organisms like yeasts and humans^16,22^. Yeasts have one α subunit Snf1, one γ subunit Snf4 and three β subunits (Sip1, Sip2, and Gal83). Humans, on the other hand, have two α subunits (α1 and α2), two β subunits (β1 and β2), and three γ subunits (γ1, γ2 and γ3). These subunits can make different heterotrimeric combinations to generate different AMPK complexes, which may have different tissue distribution, subcellular localization, and functions^22^. In other model organisms, AMPK does not seem to be essential at cellular level under standard growth conditions, although its inactivation could lead to embryonic lethality or developmental arrest^29,30^. In contrast, AMPK is essential in *T. gondii*, as demonstrated by the severe growth defects of the AMPKγ depletion mutant and AMPKα deletion mutant (Fig. 2 and Fig. S1). This highlights the differences of AMPK function between *Toxoplasma* parasites and other organisms. Among the eukaryotes whose genomes have been sequenced, the vast majority have genes encoding AMPK. Yet there are a few exceptions and malaria parasites in humans and rodents such as *Plasmodium falciparum* and *Plasmodium berghei* are examples. On the other hand, *Plasmodium* species encode a kinase called KIN that has limited sequence similarity to AMPKα^31^. The phosphomimetic mutant of KIN is able to rescue the *Snf1* deletion mutant in yeast, indicating functional conservation of these proteins. In addition, although malaria parasites do not seem to have canonical AMPKβ or AMPKγ, they do respond to salicylate that binds AMPKβ and promotes AMPKα phosphorylation, suggesting that they may have cryptic AMPK subunits that are divergent from canonical AMPKs^31^. *Toxoplasma* also encodes a KIN that is highly similar to *Plasmodium* KIN and its function remains to be elucidated. The phenotype score (derived from a genome-wide CRISPR screen that estimated the fitness cost of inactivating a given gene) for *TgKIN* (0.04) implies that it is probably not required for parasite growth under standard conditions^32^, similar to KIN in *Plasmodium* sp.

During the lytic cycle of *Toxoplasma* tachyzoites, AMPKα phosphorylation at the T221 residue is greatly increased right after the parasites are released from host cells. However, the exact signals that trigger the phosphorylation, as well as the kinases that mediate the phosphorylation are currently unknown. In mammalian cells, a variety of conditions that deplete cellular energy (drop of ATP and rise in AMP or ADP) or glucose level result in AMPKα phosphorylation and activation^13,16,22^. LKB1 is the major kinase responsible for AMPKα activation under these conditions^33,34^. In addition, AMPK may also be activated by Ca^2+^/calmodulin-dependent protein kinase (CaMKK) in response to increased cellular Ca^2+^ ^35,36^. Through co-purification with SNF1 subunits, kinases that activate yeast SNF1 were also identified^37^. In both yeasts and mammalian cells, multiple kinases are involved in AMPK activation but the homology among those kinases is largely confined to the catalytic domains. However, yeast SNF1 kinases like Tos3p can phosphorylate mammalian AMPK^37^. Likewise, LKB1 and CaMKK can function in yeasts as Snf1-activating enzymes^35,37^. These results suggest a functional conservation of the kinases that phosphorylate AMPK from different species. However, sequence analyses suggested that none of the kinases that phosphorylate AMPKα in human or yeasts seem to have orthologs in *Toxoplasma*. On the other hand, given the fact that the phosphorylation of TgAMPKα is induced following parasites egress from host cells, it is reasonable to predict that CDPKs and AGC kinases (protein kinase A, G, and C families) may be involved in phosphorylating TgAMPKα^27,38–40^. In support, some of these kinases, including CDPK6, CDPK2A, PKG, CDPK1 and CDPK3 were identified as potential AMPK interaction partners in the co-IP experiments (Supplementary data 1). Nonetheless, all these possibilities require further investigations for confirmation. It is also worth mentioning that there may be more than one kinase to activate TgAMPKα. *T. gondii* parasites have a complex life cycle. TgAMPK may sense different signals at different developmental stages and regulate different cellular activities. We found that in tachyzoites, the release of parasites into extracellular environment is accompanied by TgAMPK activation. Yet the exact trigging signal is currently unknown. We have tested a few possibilities, such as the change of Na^+^ and K^+^ concentrations that mimic intracellular and extracellular buffer conditions, Ca^2+^ level changes (through use of calcium ionophore A23187 or chelator BAPTA-AM). But none of these seemed to have a strong impact on egress induced TgAMPK activation. Therefore, further work is needed to figure out the exact signal that activates TgAMPK under this setting.

In addition to activation by phosphorylation, subcellular localization also plays a key role in regulating AMPK activity. In higher eukaryotes, AMPK shuttles between the nucleus and cytoplasm in a Ran GTPase-dependent manner and such shuttling can be modulated by stresses^41^. Our results showed that phosphorylation and activation of *Toxoplasma* AMPK was drastically increased upon the release of intracellular parasites into extracellular environments. As such, we also tested the subcellular localization of TgAMPK subunits under these two conditions. Using the AMPKα-mAID and AMPKγ-mAID strains that contained an HA tag to the C-termini of endogenous AMPKα or AMPKγ, IFA analyses showed that both AMPKα and AMPKγ were largely in the cytoplasm in intracellular parasites. However, in mechanically released extracellular parasites, nuclear AMPKα and AMPKγ could be observed in 50% and 85% of the parasites, respectively (Fig. S11), suggesting that nucleocytoplasmic shuttling of AMPK may be another mechanism to regulate AMPK activity during the lytic cycle of tachyzoites. Consistent with this observation, our co-IP experiments identified proteins involved in nuclear transport as potential binding partners of AMPK. In particular, TGGT1_243960 and TGGT1_222380 were identified in all three TgAMPK subunits-based co-IPs (table S1). TGGT1_243960 contains a nuclear transport factor 2 (NTF2) domain that is conserved in Ran (Ras-related nuclear protein) binding proteins, which are involved in the transport of macromolecules between the cytoplasm and nucleus, as well as the nucleocytoplasmic shuttling of AMPK. TGGT1_222380 is an ortholog of mammalian exportin 7 and exportins are known to mediate nuclear export of AMPK in higher eukaryotes. Whether these two proteins are involved in the nucleocytoplasmic shuttling of AMPK in *Toxoplasma* parasites, and the physiological significance of such shuttling are open questions to be addressed in the future. In addition, AMPK containing complexes are also found at other organelles such as lysosomes, mitochondria, and endoplasmic reticulum in higher eukaryotes^26^. The lysosomal complex containing aldolase, v-ATPase, Ragulator, axin and LKB1 activates AMPK during glucose starvation before decrease of ATP occurs^42^. More severe metabolic stresses are required to activate cytosolic and mitochondrial pools of AMPK^26^. Our localization results showed than *Toxoplasma* AMPK is mainly in the cytoplasm, but the possibility of localization in other organelles could not be excluded. In particular, although *Toxoplasma* does not seem to encode Ragulator and axin, fructose-1 6-bisphosphate aldolase (ALD), which mediates glucose sensing by the lysosomal AMPK complex, was also found to be a potential interaction partner of TgAMPK (Supplementary data 1). Whether the parasite ALD forms a complex with AMPK and has a similar glucose sensing function remains to be determined.

In other model organisms, AMPK regulates a wide variety of targets to control cellular homeostasis, through transcriptional and posttranslational modification approaches. The list of AMPK targets is still growing^43^. From our results of over-expressing different alleles of TgAMPKα in the TgAMPKγ depletion mutant, it is clear that the phosphorylation status of TgAMPKα at the T221 position has significant impacts on anabolism and catabolism. But the underlying mechanisms are largely unknown. It is also not clear whether the overexpressed TgAMPKα in the absence of TgAMPKγ used the same mechanism to regulate parasite metabolism and activities as native TgAMPKα (such as that in wildtype parasites) in the presence of TgAMPKγ, because in other model organisms, the activation and subsequent activities of AMPK may be modulated differently by different degree and types of stress or metabolic fluctuations^44^. Nonetheless, TgAMPKγ depletion leads to growth arrest but does not seem to cause dramatic alteration in gene transcription. On the other hand, it abolishes TgAMPKα phosphorylation. In our phospho-proteomic analysis, the abundance of 285 unique phospho-peptides, corresponding to 170 proteins, was significantly (*P* < 0.05, fold change > 1.5) changed after TgAMPKγ depletion. These include many metabolic enzymes and regulators, such as pyruvate kinase 1, fructose-1,6-bisphosphate aldolase, glucose transporter GT1 and pyruvate dehydrogenase kinase that are involved in sugar breakdown and ATP production^45–47^, as well as a number of eukaryotic initiation factors and CDP-alcohol phosphatidyltransferase that are involved in the synthesis of proteins and phospholipids. Many of these proteins were also identified in the co-IP experiments as potential interaction proteins with AMPK. These proteins are possible targets of AMPK in *Toxoplasma*. Further studies are needed to dissect how exactly they are regulated by AMPK and the physiological significance of such regulation. On the other hand, when some of the differentially phosphorylated proteins listed in Fig. 7 (such as GT1, ALD, PYK1 and ACS) were analyzed in detail to see whether the residues potentially phosphorylated by TgAMPK were conserved among other organisms. Interestingly, while orthologs of these proteins are present in model eukaryotes from yeasts to fruit flies and humans, the phosphorylated residues within them are either not conserved or do not have evidence of AMPK-dependent phosphorylation. These results suggest that either the proteins analyzed are not direct substrates of TgAMPKα, but other kinases whose activities are influenced by TgAMPKα, or the detailed mechanisms by which *Toxoplasma* AMPK regulates parasite metabolism are different from that of other eukaryotes.

## Methods

### Parasite cultures

Tachyzoites of the RH *Δhxgprt*^3^ and RH *∆hxgprt* Tir1 strains^27^ and their derivatives were propagated in human foreskin fibroblasts (ATCC, USA), which were cultured in Dulbeccos’s modified Eagles medium (DMEM) (Sigma-Aldrich, USA) supplemented with 2% fetal bovine serum (FBS) (Gibco, USA), 2 mM glutamine, and 1% penicillin–streptomycin. All strains used in this study are listed in Table S2 in the *Supplementary information*. Unless otherwise indicated, parasites were forced to egress from host cells by sequential syringe passage through 22-gauge needles and filtered by filtration through 3-µm polycarbonate membranes (Whatman, USA) before use.

### Identification of putative AMPK subunits in *Toxoplasma* by bioinformatic analyses

To identify orthologues of the AMPK complex in *Toxoplasma*, sequences of human (α2, β1 and γ2) AMPK and yeast SNF1 (Saccharomyces cerevisiae Snf1, Gal83 and Snf4) were used as query sequences to perform protein BLAST searches against *Toxoplasma* genome in ToxoDB (www.toxodb.org). To identify TgAMPKα, *Toxoplasma* hits from human AMPKα2 and yeast Snf1 based BLAST searches with an E value below 1e-50 were used as query sequences to BLAST against the human and Saccharomyces cerevisiae genomes, respectively. Only the hits that identified human AMPKα and yeast Snf1 as the top match in the reciprocal BLAST searches were accepted as putative AMPKα subunits in *Toxoplasma*. A similar approach was used to identify TgAMPKβ and TgAMPKγ, except that original hits (performed in ToxoDB using human β1, γ2 and yeast Gal83, Snf4 as baits) with E values below 0.001 were subjected to reciprocal BLAST searches in human and yeast.

### Construction of plasmids and transgenic parasite strains

All plasmids used in this study, as well as the methods for their construction, are listed in Table S3 in the *Supplementary Information*. All primers used were synthesized by a commercial provider (Tsingke Biotechnology Co.,Ltd, Beijing, China) and are listed in Table S4. All plasmids were constructed by multi-fragment cloning using the ClonExpress MultiS Cloning Kit (Vazyme Biotech, China) except the locus specific CRISPR plasmids, which were generated by replacing the *UPRT*-targeting gRNA sequence in pSAG1-Cas9-U6-sgUPRT with locus-specific guide sequences, following previously described protocols^48^. All transgenic strains were constructed by CRISPR/Cas9-mediated site-specific gene editing^49^.The AMPKγ-mAID strain was constructed by co-electroporating the pSAG1::Cas9-U6::sg-AMPKγ-3UTR plasmid and the AMPKγ-mAID~HXGPRT fragment derived from the pUC19-AMPKγ-mAID~HXGPRT plasmid into purified tachyzoites of the RH *∆hxgprt* Tir1 strain. Transfectants were selected with 25 µg/ml mycophenolic acid (MPA) and 50 µg/ml xanthine (Sigma-Aldrich, USA) and single cloned by limiting dilution. Individually clones were screened by diagnostic PCRs (primers are listed in Table S4) and immunofluorescent assays (detecting the presence of HA tag). Subsequently, a final concentration of 500 µM indole-3-acetic acid (IAA) (Sigma-Aldrich, USA) was used to induce the degradation of AMPKγ in the AMPKγ-mAID strain. The AMPKα-mAID strain was constructed in the same way. The AMPKγ complementation strain Comp-γ, as well as the AMPKα-T221T, -T221D, or -T221A expressing strains were constructed by co-transfecting the AMPKγ or AMPKα expressing cassettes and the *UPRT* targeting pSAG1‐Cas9‐sgUPRT into tachyzoites of the AMPKγ-mAID strain. Transfectants were selected with 10 µM 5-fluoro-2'-deoxyruridine (FUDR) (Sigma-Aldrich, USA) and 1 µM pyrimethamine (Sigma-Aldrich, USA) and single clones were examined by diagnostic PCRs and IFA before use. The RH *Δhxgprt*/LoxP-*AMPKα-TY* strain was constructed by co-transfecting pSAG1:Cas9-sgAMPKα and the Loxp-AMPKα-TY containing fragment amplified from pTUB1-Loxp-AMPKα-TY-LoxP-YFP-DHFR into the RH *Δhxgprt* strain and selected with 1 µM pyrimethamine. Drug resistant transfectants were single cloned by limiting dilution. The RH *Δhxgprt*/LoxP-*AMPKβ-TY* strain was generated in a very similar way. In both cases, single clones were examined by diagnostic PCRs and IFA before use.

### Co-immunoprecipitation (Co-IP) to identify the proteins interacting with each of the *Toxoplasma* AMPK subunits

Fresh tachyzoites of the transgenic strains RH *Δhxgprt*/LoxP-AMPKα-TY, RH *Δhxgprt*/LoxP-AMPKβ-TY or AMPKγ-mAID, as well as the corresponding parental strains (RH *Δhxgprt* for AMPKα and AMPKβ, RH *∆hxgprt* Tir1 for AMPKγ) that were used as controls, were needle released from host cells and purified by filtration through 3 µm polycarbonate membranes. Then 5 × 10^8^ parasites from each strain were collected, washed three times with ice cold PBS, and lysed with 400 μl Western and IP lysis buffer (Beyotime, Shanghai, China) containing phenylmethylsulfonyl fluoride. During lysis, the samples were placed in a water bath sonicator and sonicated for 30 min in ice water. The lysates were then centrifugated at 12,000 g for 10 min and the supernatants were collected for subsequent co-IP experiments. To do co-IP, the supernatants of the parasite lysates were cleared by incubation with mouse IgG conjugated protein AG agarose beads (Beyotime, Shanghai, China) at 4 °C for 2 h, to remove proteins that bind to agarose beads or mouse IgG. The cleared supernatants were then added to protein AG agarose beads conjugated with mouse anti-Ty antibodies or mouse anti-HA antibodies (Medical & biological laboratories CO., LTD, Tokyo, Japan) and incubated overnight at 4 °C. Subsequently, the beads were pelleted by centrifugation at 1000 g for 2 min and washed with ice cold PBS three times to remove unbound proteins. Finally, the bound proteins were subjected to on-bead trypsin digestion and then identified by mass spectrometry, following the protein identification methods described below in the LC-MS/MS and data analysis sections.

### Immunofluorescent assays (IFA) and Western blotting (WB)

IFAs and Western Blots that examined protein expression were performed according to established protocols^45,48^. The following antibodies were used: rabbit anti *Toxoplasma* aldolase (1:5000 dilution for WB and 1:2000 for IFA), mouse anti Ty tag (the BB2 clone of anti-Ty1, provided by Prof. David Sibley, Washington University in St. Louis, USA) (1:2000 for WB and 1:1000 for IFA), mouse anti HA tag (the TANA2 clone of anti-HA, MBL International Corporation, Japan) (1:1000 for WB and 1:300 for IFA), mouse anti *Toxoplasma* SAG1 (provided by Prof. David Sibley, Washington University in St. Louis, USA) (1:2000 for IFA), mouse anti *Toxoplasma* GRA1 (1:5000 for WB), rabbit anti *Toxoplasma* AMPKα (produced by immunizing rabbits with an AMPKα derived peptide (GSPKDPSRSPGRSE) conjugated to keyhole limpet hemocyanin) (1:1000 for WB), rabbit anti phospho-AMPKα(Thr172) (Beyotime, China) (1:500 for WB), IRDye 680RD Goat anti-Rabbit IgG (1:5000 for WB), IRDye 800CW Goat anti-Mouse IgG (LI-COR Biosciences. USA) (1:5000 for WB), HRP-labeled Goat Anti-Rabbit IgG (Beyotime Biotechnology, China) (1:1000 for WB), Alexa Fluor 488 conjugated Goat anti-Mouse IgG (1:2000 for IFA), Alexa Fluor 594 conjugated Goat anti-Mouse IgG (1:2000 for IFA), Alexa Fluor 488 conjugated Goat anti-Rabbit IgG (1:2000 for IFA) and Alexa Fluor 594 conjugated Goat anti-Rabbit IgG (1:2000 for IFA) (Fisher Scientific. USA). IFA images were acquired by an Olympus BX53 microscope (Olympus Life Science, Japan) equipped with an Axiocam 503 mono camera (Carl Zeiss Inc., Germany) and a ZEN 2 (blue edition) software (Carl Zeiss Inc., Germany). Western Blots were scanned by an Amersham Typhoon 5 imager (GE healthcare, UK). Uncropped and unprocessed IFA and Western blotting images were provided in the Source Data file, as well as in the Supplementary Information.

### Assessments of parasite fitness in vitro

Plaque assays that estimate the overall growth of parasites in HFF monolayers, two-color invasion assay that determines the invasion efficiency of parasite into HFF cells, motility assay that examines the gliding of purified parasites on BSA coated coverslips, as well as the intracellular replication assay that determines the proliferation efficiency in HFF cells were performed following previously described protocols^45,48,50^. All assays were independently repeated twice or three times, each with three biological replicates. To test the impact of AMPK inhibitor and activator on parasite invasion, 20 μM A769662 (activator) (Macklin Inc., Shanghai, China) or 5 μM compound C (inhibitor) (MedChemExpress LLC, Shanghai, China) were used to treat RH parasites for 1 h before egress. Then the parasites were released by needle passage, purified by filtration, and used to infect HFF cells for 20 min. Subsequently a two-color staining protocol was used to determine the invasion efficiency. To test the effects of the two compounds on parasite replication, 20 μM A769662 or 5 μM compound C were used to treat MEF/AMPKα1^-^ AMPKα2^-^ cells (provided by Dr. Chen-Song Zhang from Xiamen University) infected with RH parasites for 24 h. Then the number of parasites in each PV was determined by IFA, as previously described^45^.

### Determine the ATP level in parasites

Cellular ATP levels were examined by a commercial ATP Assay Kit (Beyotime, China), which quantifies the ATP level by determining the intensity of luminescence generated by the ATP dependent enzyme firefly luciferase. Freshly harvested parasites (5 × 10^6^) were lysed in 100 μL lysis buffer on ice for 30 min and centrifuged at 12,000×g for 5 min. The supernatant was collected for subsequent examination. To detect the ATP levels, 100 μL of each sample or serially diluted ATP standard solution (5 μM, 2.5 μM, 1.25 μM, 0.625 μM, 0.312 μM and 0.078 μM) was mixed with 100 μL of ATP detection working solution in opaque 96-well plates and the bioluminescence signal in each well was determined in the Cytation5 plate reader (Biotek, USA).

### Nascent protein synthesis

The rates of nascent protein synthesis in parasites were evaluated following a previously described method^51^. Invaded parasites were grown in DMEM with or without IAA for 35 h. Then the cultures were washed with pre-warmed PBS and cultured in methionine-free DMEM medium for 30 min. Subsequently, 100 μM L-homopropargyl glycine (APExBIO, USA) was added and incubated for 6 h. After treatment, the cultures were harvested and the parasites were needle released from host cells, fixed the with 4% paraformaldehyde for 15 min, permeabilized with 0.1% Triton X-100 for 10 min, blocked with 10% fetal bovine serum for 30 min, and washed with PBS. Samples were then resuspended in the CuAAC reaction solution (200 μM TBTA, 400 μM TECP, 200 μM CuSO4). 10 μM FAM azide 5-isomer (APExBIO, USA) was then added to each sample and incubated at 4 °C overnight with shaking. Parasites were collected by centrifugation at 12,000×g for 5 min, stained with Hoechst (Beyotime, China) for 20 min in dark, washed extensively with PBS, and eventually resuspend in 300 μL PBS. The fluorescence intensity of parasites in each sample was analyzed by a cytoflex-LX flow cytometer (Backman, USA), with > 10,000 parasites being analyzed for each sample. Each strain and condition were tested three times independently.

### RNA-Seq analysis

Intracellular parasites of the AMPKγ-mAID strain were treated with or without IAA for 48 h prior to egress. Then the parasites were released by needle passage and purified by filtration to remove host debris. Subsequently, total RNA was extracted from the parasites using the TransZol Up Reagent according the manufacturer’s instructions (TransGen Biotech, China). The quality of RNA samples was assessed by the Agilent 2100 Bioanalyser (Agilent, USA). Library construction was done with the TruseqTM RNA sample prep Kit (Illumina, USA). RNA sequencing was performed on a HiSeq xten/NovaSeq 6000 sequencer (Illumina, USA). Then, the clean reads were mapped onto the genome of the GT1 strain using the Hisat2 software (HISAT2 (daehwankimlab.github.io)). Expression of genes was profiled by the values of transcripts per million reads (TPM) through RSEM v1.3.1 (http://deweylab.biostat.wisc.edu/rsem/), and differentially expressed genes were identified by comparing their TPM values using DESeq2 v1.24.0 (http://bioconductor.org/packages/stats/bioc/DESeq2/). Genes with *p*-value < 0.05 and fold change ≥ 2 (with IAA vs without IAA treatment) were treated as differentially expressed genes. The experiment was repeated three times independently and the raw data have been deposited to the SAR database (https://submit.ncbi.nlm.nih.gov/) under the accession number PRJNA782309.

### Proteomic and phosphoproteomic sample preparation

The AMPKγ-mAID strain was pretreated with or without IAA for 48 h, followed by needle passage to break host cells and release parasites. Tachyzoites were then collected, purified by filtration, and lysed in 8 M urea (0.1 M Tris-Cl, pH 8.5) with the protease inhibitor cocktail (Merk, USA) and phosphatase inhibitor cocktail (Roche Applied Science, Germany). Three samples were prepared independently for each strain under each condition. The lysate was sonicated and centrifuged for 15 min at 4°C to collect supernatant for protein concentration assessment using the BCA assay (Thermo Scientific, USA). Then 500 μg protein of each sample was used for digestion. TCEP (Tris(2-carboxyethyl) phosphine, final concentration is 5 mM) (Thermo Scientific, USA) and iodoacetamide (final concentration is 10 mM) (Sigma-Aldrich, USA) for reduction and alkylation were added to protein samples and incubated at room temperature for 30 min, respectively. The protein mixture was diluted four times (to reduce the concentration of urea to 2 M) and digested at 37 °C with Lys-C at 1:100 (w/w) (Wako, Japan) for 2 h, and then with trypsin at 1:50 (w/w) (Promega, USA) overnight. Then the reaction was stopped by adding formic acid to pH <2.

Digested samples were desalted by a SepPak tC18 3cc Vac cartridge (Waters Corporation, USA) and dried out by lyophilization. The samples were then resolved in 100 mM HEPES (pH = 8.0), and the peptide concentration was measured using the Quantitative Colorimetric Peptide Assay (Thermo Scientific, USA). 100 μg peptide of each sample (six samples in total, three with IAA treatment and three without) was labeled using the six-plex TMT kit (Thermo Scientific, USA) following the manufacturer’s manual. Sample labeling efficiency of each channel was checked as previously described^52^. The labeling reaction was quenched by hydroxylamine and then desalted by the tC18 Vac cartridge as described above.

After TMT labeling, all peptides from the six samples were mixed (600 μg in total) and 30 μg was used for proteomic analysis. The samples were subject to high pH reversed-phase peptide fractionation (Pierce, USA) and 13 fractions were acquired for LC-MS analyses. The rest of labeled peptides (570 μg in total) were used for phospho-peptide enrichment using a SMOAC workflow, which enriched phospho-peptides sequentially by TiO_2_ and IMAC (Thermo Scientific, USA).

### LC–MS/MS analysis

For proteomic analysis, the 13 fractions were analyzed by a home-made 30 cm-long pulled-tip analytical column (100 μm ID x 360 μm OD, ReproSil-Pur C18-AQ 1.9 μm resin, Dr. Maisch GmbH, Germany). The column was then placed in-line with an Easy-nLC 1200 nano HPLC (Thermo Scientific, USA) that was connected to the mass spectrometer. The analytical column temperature was set at 55°C during the experiments. A binary buffer system (buffer A: 0.1% formic acid in water; buffer B: 0.1% formic acid in 80% acetonitrile) with a 180-min gradient (5–10% buffer B over 1 min; 10–40% buffer B over 145 min; 40–60% buffer B over 20 min; 60–100% buffer B over 1 min; 100% B for 13 min). MS data were acquired by Q-Exactive mass spectrometer (Thermo Scientific, USA) in a data-dependent top-20 method with a maximum injection time of 50 ms, a scan range of 300–1800 m/z, and an AGC target of 3 × e^6^. MS/MS was performed via higher energy collisional dissociation fragmentation with a target value of 1 × e^5^ and a maximum injection time of 100 ms. Full MS and MS/MS scans were acquired at a resolution of 70,000 and 17,500, respectively. Dynamic exclusion was set to 30 s. The proteins co-precipitated with each of the *Toxoplasma* AMPK subunits were identified by the same LC–MS/MS approach described in this section.

For phosphopeptide analysis, each enriched peptide sample was split into two parts. One half was used in an online two-dimensional LC separation called MudPIT (Multidimensional Protein Identification Technology) as described previously^53^. The other half was loaded on a single reverse phase column with two technical replicates. For the MudPIT, enriched peptides were loaded onto a biphasic pulled-tip column (75 μm ID x 360 μm OD), home packed with 20 cm C18 resin (ReproSil-Pur C18-AQ 1.9μm resin, Dr. Maisch GmbH, Germany) followed by 5 cm SCX resin (5 μm, Phenomenex, USA). The column was then placed in-line with an Easy-nLC 1200 nano HPLC (Thermo Scientific, USA) for mass spectrometry analysis. Peptides were auto injected into the SCX phase of the biphasic column, and unbound peptides were analyzed by the analytical C18 column. Then, the SCX bounded peptides were sequentially eluted with 5%, 15%,20%, 25%, 30%, 50%, 100% of 500 mM ammonium acetate. Each fraction was analyzed by the reverse phase column with a 120-min gradient (buffer A: 0.1% formic acid in water; buffer B: 0.1% formic acid in 80% acetonitrile): 5–35% buffer B over 95 min; 35–60% buffer B over 10 min; 60–100% buffer B over 2 min; 100% buffer B over (13 min). MS data were acquired by Q-Exactive mass spectrometer (Thermo Scientific, USA) in a data-dependent top-20 method with a maximum injection time of 50 ms, a scan range of 300–1800 m/z, and an AGC target of 3 × e^6^. MS/MS was performed via higher energy collisional dissociation fragmentation with a target value of 5 × e^5^ and a maximum injection time of 100 ms. Full MS and MS/MS scans were acquired at a resolution of 70,000 and 17,500, respectively. Dynamic exclusion was set to 30 s. For the single reverse phase analyses, the MS method was the same as the MudPIT method except a 180-min gradient (5–35% buffer B over 145 min; 35–60% buffer B over 20 min; 60–100% buffer B over 2 min; 100% buffer B over 13 min) was run. The mass spectrometry data generated in this study have been deposited to the ProteomeXchange Consortium (http://proteomecentral.proteomexchange.org) via the PRIDE partner repository with the identifier number PXD039148.

### Data analysis

The acquired MS/MS data were analyzed against a *Toxoplasma gondii* database (ToxoDB.org) by Maxquant V1.6.10.43 using the default settings. Tolerance of precursor mass and fragment mass were set to 20 ppm. The main search peptides tolerance was set at 4.5 ppm according to the feature of the instrument used in the study. Carbamidomethylation of cysteine (+57.021 Da) was set as static modifications. The quantification search type chose the reporter ion ms2 using the TMT 6plex method. Oxidation of methionine (+15.995 Da) and acetylation of protein N terminus were set as variable modifications for the proteomic data analysis, and phosphorylation of Serine/Threonine/Tyrosine (+79.9663 Da) for the phospho-proteomic data. Trypsin was defined as cleavage enzyme at full specific mode, and the maximum missing cleavage was set at 2. FDR of the identified proteins was set at 1%, which was calculated at the peptide level.

The interpretation of proteomic results from MaxQuant was done in the R4.0.0. platform (https://www.r-project.org/). The tidyverse package (https://www.tidyverse.org/packages/) was used to prepare data, and the *limma* package (https://bioconductor.org/packages/release/bioc/html/limma.html) was used to fit the expression data and calculate the logarithmic fold change and *p*-values. The sum of reporter intensity values (calculated by Maxquant) of all unique peptides matching a given protein was used to compare its abundance among samples. Proteins with log_2_ (fold change) ≥ 0.58 (fold change ≥ 1.5), and *p*-value ≤ 0.05 were considered as differentially expressed. The interpretation of phosphoproteomic results was done in the Python 3 (https://www.python.org/download/releases/3.0/), and Perseus (https://maxquant.net/perseus/) Platforms. The PaDuA package in the Python 3 environment was used for data cleaning^54^, data quality summary and visualizing. Persues (v1.6) was used to add linear motif and the preprocessCore package was used to normalize the phosphopeptide intensity^55^. The *limma* package was used to fit the expression data and calculate the logarithmic fold change and *p*-values. The abundance change of each phosphopeptide was normalized by the fold change (FC) of corresponding proteins. For proteins that had a significant abundance change (protein *p* ≤ 0.05), the differentially phosphorylated peptides were called if the FC of phosphorylation / FC of protein abundance ≥ 1.5 and the phosphopeptide *p*-value was ≤ 0.05. For proteins that did not have a significant abundance change (*p* > 0.05), phosphorylation sites with log_2_ (phospho fold-change) ≥ 0.58 and *p*-value ≤ 0.05 were considered as differentially phosphorylated.

### Statistics and reproducibility

Except for the transcriptomic and proteomic data, all other results were analyzed with Prism 8 (GraphPad Software Inc., USA). Sample sizes were chosen according to similar experiments published in previous literatures. Statistical analyses were performed by unpaired two-tailed Student’s t-test or two-way ANOVA with Tukey’s multiple comparisons post-tests, as indicated in figure legends. All IFA or Western blotting results were repeated at least twice using independently prepared samples and similar results were obtained. Only representative images were shown.

### Reporting summary

Further information on research design is available in the Nature Portfolio Reporting Summary linked to this article.

## Supplementary information

Supplementary Information

Description of Additional Supplementary Files

Supplementary Dataset 1

Supplementary Dataset 2

Supplementary Dataset 3

Supplementary Dataset 4

Reporting summary

## Data Availability

The RNA-Seq data generated in this study have been deposited in the SRA database under accession code PRJNA782309. The phospho-proteomic data generated in this study have been deposited in the ProteomeXchange Consortium (http://proteomecentral.proteomexchange.org) via the PRIDE partner repository with the identifier number PXD039148. All other data are presented in the article or the supplementary information. Source data are provided with this paper.

## Source data

Source Data
