# Supplementary material for: Rapid metabolic reprogramming mediated by the AMP-activated protein kinase during the lytic cycle of Toxoplasma gondii
Source: Nat Commun. 2023 Jan 26;14:422. doi: 10.1038/s41467-023-36084-0 (PMC9880002; doi:10.1038/s41467-023-36084-0)
Supplement: Supplementary file 1 — Supplementary Information [file 41467_2023_36084_MOESM1_ESM.pdf]

Supplementary Information for

Rapid metabolic reprogramming mediated by the AMP-activated protein kinase during the lytic cycle of *Toxoplasma gondii*

Yaqiong Li [1], Zhipeng Niu [1 #], Jichao Yang [1 #], Xuke Yang [1 #], Yukun Chen [1], Yingying Li [1], Xiaohan Liang [1], Jingwen Zhang [1], Fuqiang Fan [1], Ping Wu [2], Chao Peng [2], Bang Shen [1, 3, 4 *]

[1] State Key Laboratory of Agricultural Microbiology, College of Veterinary Medicine, Huazhong Agricultural University, Wuhan, Hubei Province, 430070, PR China.

[2] National Facility for Protein Science in Shanghai, Zhangjiang Lab, Shanghai Advanced Research Institute, Chinese Academy of Science, Shanghai, 201210, PR China.

[3] Key Laboratory of Preventive Medicine in Hubei Province, Wuhan, Hubei Province, 430070, PR China.

[4] Hubei Hongshan Laboratory, Wuhan, Hubei Province, 430070, PR China.

[#] Equal contributions

[*] Corresponding author: Bang Shen

Email: shenbang@mail.hzau.edu.cn

This PDF file includes:

Figures S1 - S11

Tables S1 – S4

Supplemental References

Unprocessed blots and uncropped gels for supplementary figures

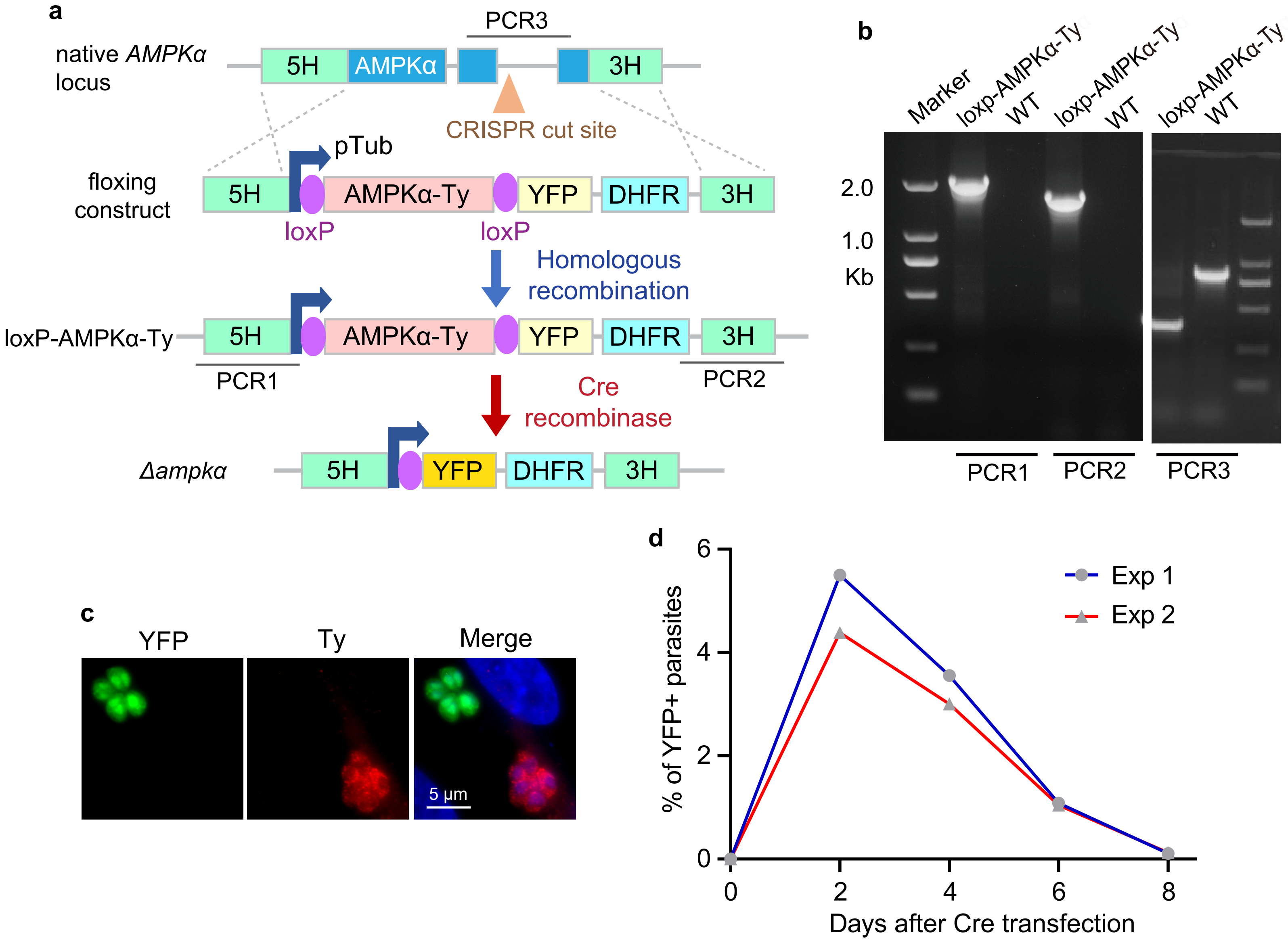

Fig S1. Construction and phenotypic analyses of a loxP-AMPKα-Ty strain. **a**, CRISPR/Cas9 mediated homologous recombination replacing endogenous AMPKα with loxP flanked AMPKα to construct the loxP-AMPKα-Ty strain. Then, a plasmid expressing the Cre recombinase was transfected into the loxP-AMPK α-Ty strain to induce recombination between two loxP sites, which would delete AMPKα and move YFP close to the pTub to activate its expression. As such, YFP expression could be used as a reporter for AMPKα deletion. **b**, diagnostic PCRs on a loxP-AMPKα-Ty clone. **c**, IFA examination of YFP and AMPKα-Ty expression in the loxP-AMPKα-Ty strain 24 hours post the transfection with the Cre expression plasmid. **d**, the percentage of YFP^+ parasites in the population after transfecting the Cre expressing plasmid into the loxP-AMPKα-Ty strain, as determined by flow cytometry at indicated time points. The two curves represent n=2 independent experiments. Source data are provided as a Source data file.

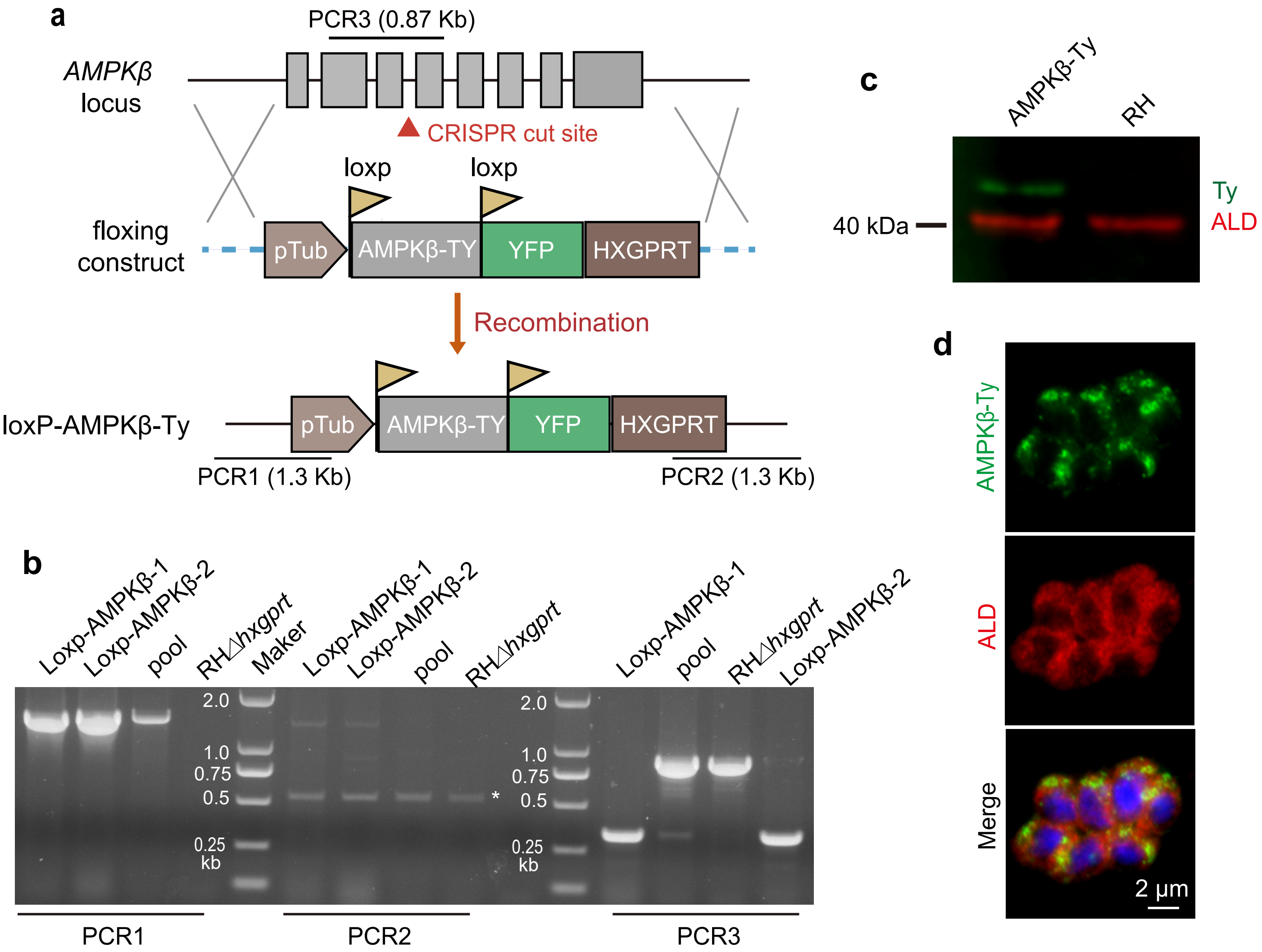

Fig S2. Construction and verification of a RH loxP-AMPKβ-Ty strain used for Co-IP to identify proteins interacting with AMPKβ. **a**, schematic illustration of loxP-AMPKβ-Ty strain construction using CRISPR/Cas9 assisted recombination to replace endogenous AMPKβ with a Ty tagged and loxP flanked copy of AMPKβ. **b**, diagnostic PCRs on two loxP-AMPKβ-Ty clones and the drug resistant pool after transfection. * non-specific amplification. **c-d**, Western blotting (c) and IFA (d) to confirm the expression of Ty tagged AMPKβ in the loxP-AMPKβ-Ty strain. Source data are provided as a Source data file.

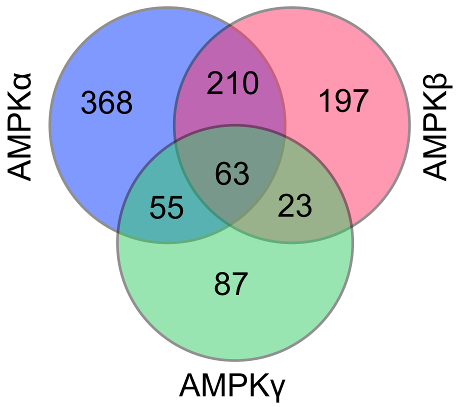

Fig S3. Venn diagram showing the overlap of proteins identified by co-IPs using different AMPK subunits as baits. For each co-IP experiment, proteins satisfying the following criteria were treated as hits: no unique peptides were found in the control group, or the number of unique peptides in the experimental group was twice or more of that in the control group. Detailed lists of the identified proteins were provided in Table S1 and Supplementary data 1.

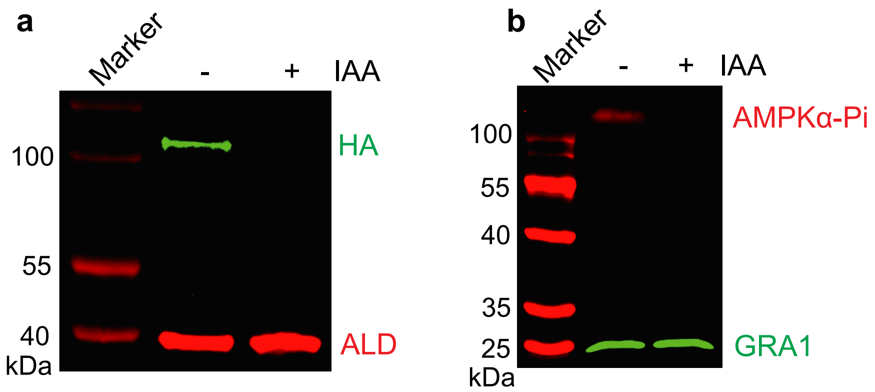

Fig S4. The phospho-AMPKα(Thr172) antibody specifically recognized TgAMPKα in *Toxoplasma* parasites, as revealed by Western blotting using the TgAMPKα depletion strain AMPKα-mAID. The AMPKα-mAID contained an auxin-inducible degron mAID (HA tagged) fused to the C-terminus of endogenous AMPKα. Therefore AMPKα could be depleted by IAA treatment. The Western blot was performed using lysates prepared from purified parasites of the AMPKα-mAID strain that were treated with (+) or without (-) IAA treatment, and probed with anti-HA (a) to examine the protein level of TgAMPKα or anti-phospho-AMPKα(Thr172) (b) to check the level of phosphorylated TgAMPKα. GRA1 and ALD were included as loading controls. Source data are provided as a Source data file.

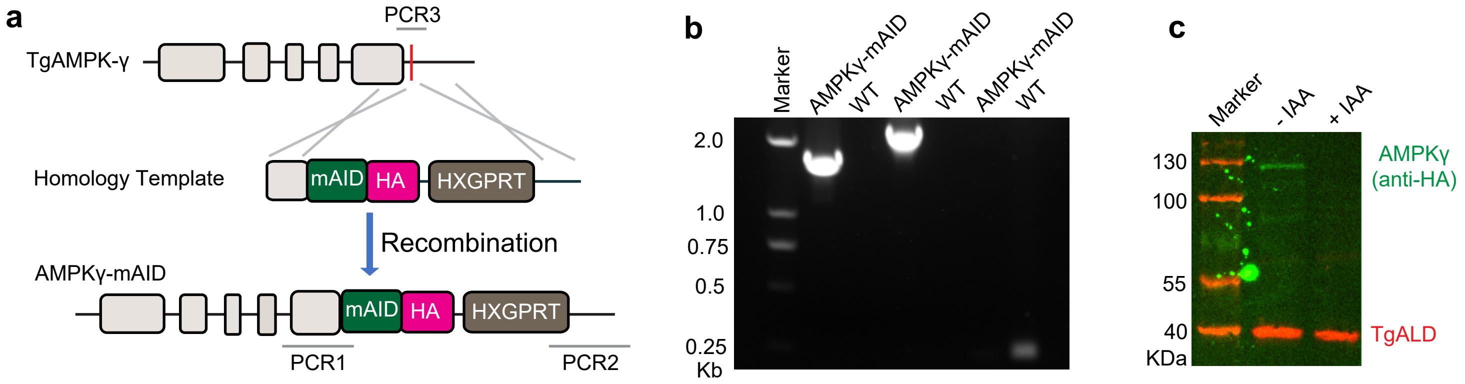

Fig S5. Construction of the AMPKγ-mAID strain for IAA induced AMPKγ depletion. **a**, schematic illustration of adding a mAID tag to the C terminus of AMPKγ at the endogenous locus, through CRISPR/Cas9 induced homologous recombination. The red bar indicates the CRISPR targeting site. **b**, Diagnostic PCR on an AMPKγ-mAID clone. **c**, Depletion of AMPKγ expression in the AMPKγ-mAID strain after 48 hours' IAA treatment, as determined by Western blotting. Source data are provided as a Source data file.

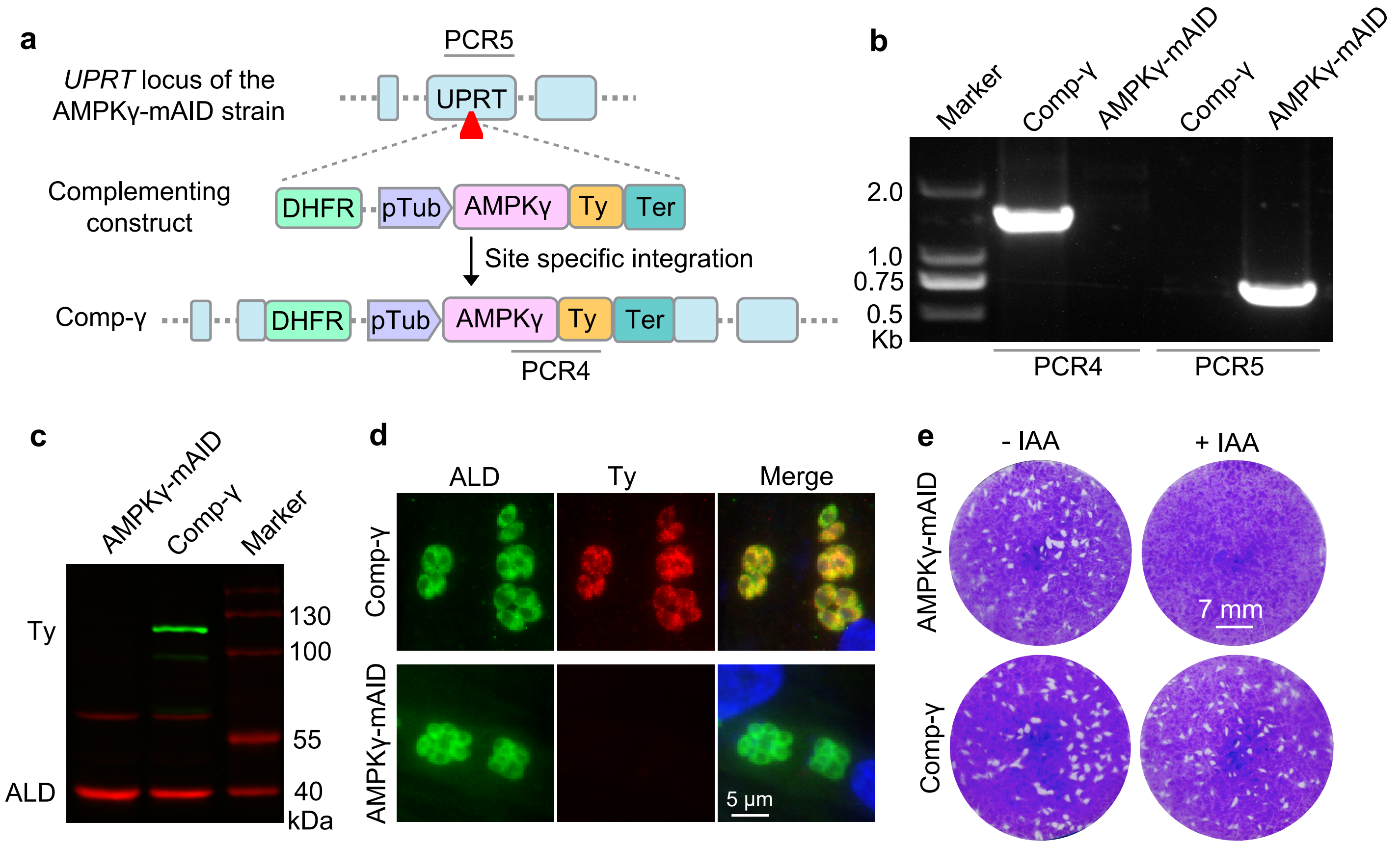

Fig S6. Construction and characterization of an AMPKγ complementation strain in the AMPKγ-mAID mutant. **a**, schematic illustration of inserting an AMPKγ expressing cassette into the *UPRT* locus of the AMPKγ-mAID mutant to construct the complementation strain Comp-γ, through CRISPR/Cas9 induced site-specific integration. The red triangle indicates the CRISPR targeting site. **b**, Diagnostic PCR on a Comp-γ clone. **c-d**, Western blotting (c) and immunofluorescent staining (d) checking the expressing of complementing AMPKγ. **e**, plaque assay examining the restore of parasite growth after AMPKγ complementation. Tachyzoites of the indicated strains were cultured in HFF monolayers for 7 days with or without IAA treatment. The plaques formed were visualized after crystal violet staining. Source data are provided as a Source data file.

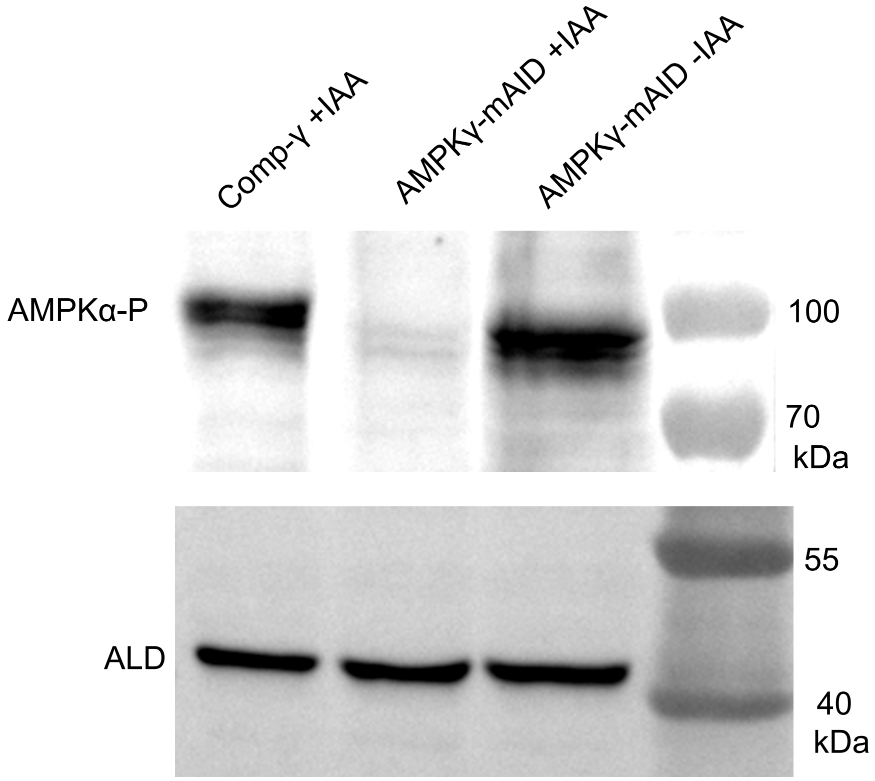

Fig S7. Restore of AMPKα phosphorylation in AMPKγ depletion mutants by AMPKγ complementation. Extracellular parasites pretreated with or without IAA were subject to Western blot analyses, using antibodies against phosphorylated AMPKα (anti phospho-AMPKα(Thr172)) and *Toxoplasma* ALD. Source data are provided as a Source data file.

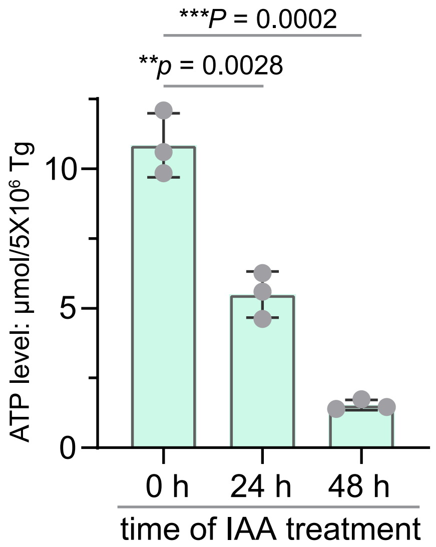

Fig S8. Decrease of cellular ATP after AMPKγ depletion. Intracellular parasites of the AMPKγ-mAID strain were treated with IAA for 0, 24 or 48 hours before harvest. Then the parasites were needle released from host cells, purified by filtration and the ATP level was determined using a bioluminescence-based ATP assay kit. The experiment was repeated n=3 times independently. Means ± SD, unpaired two-tailed Student's t-test. Source data are provided as a Source data file.

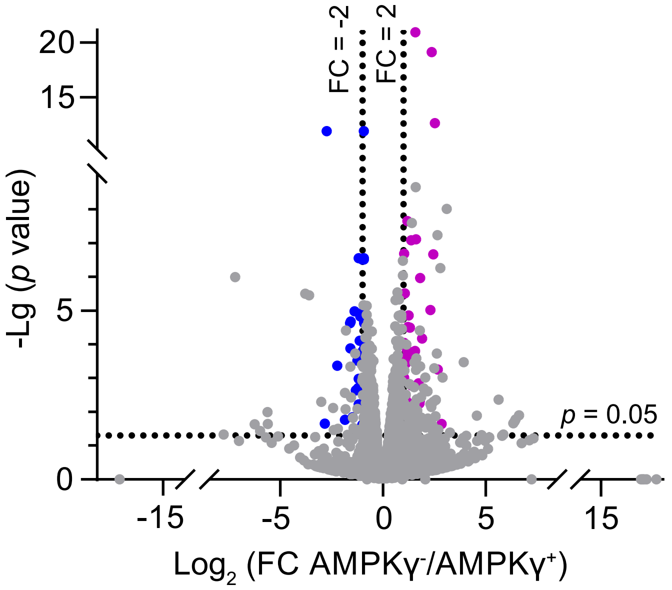

Fig S9. Volcano plot showing the gene expression change upon AMPKγ depletion. The AMPKγ-mAID parasites pretreated with or without IAA for 24 hours were subjected to RNA-Seq analysis. The fold change (FC) of each gene and the corresponding *p*-value (determined by the Wald test in DEseq2) were graphed. Each sample was examined n=3 times independently. Blue and purple dots represent differentially expressed genes, which were identified according to the following criteria: FC ≥ 2 or ≤ -2, *p*-value ≤ 0.05, the average TPM value in at least one group (AMPKγ^- or AMPKγ^+) was over 10.

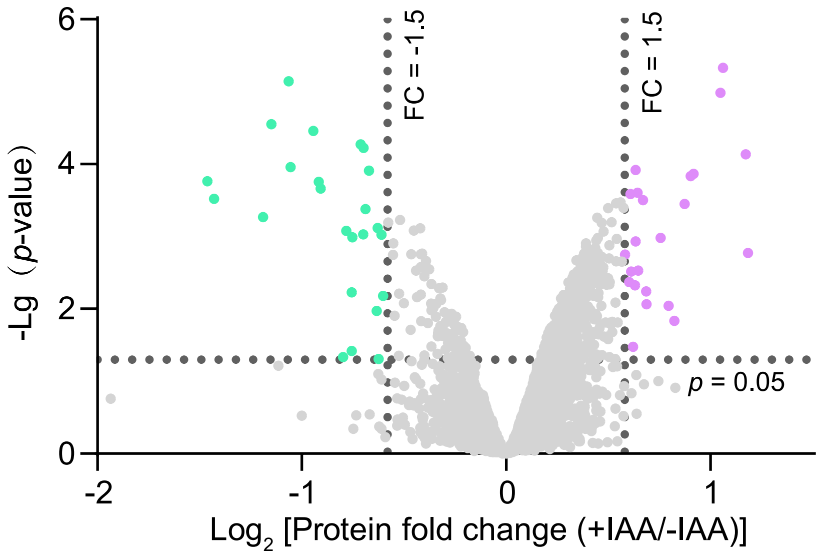

Fig S10. Protein abundance changes caused by TgAMPKγ depletion in the AMPKγ-mAID strain, as determined by quantitative proteomics. The AMPKγ-mAID parasites pretreated with or without IAA for 48 hours were released from host cells by needle passage and then subjected to LC-MS/MS analysis. This experiment was performed side by side with the phosphoproteomic experiment presented in Fig 7 and the same n=3 sets of samples were used, except that no phospho-peptide enrichment step was involved. *P* values were determined by Empirical Bayes two-tailed tests.

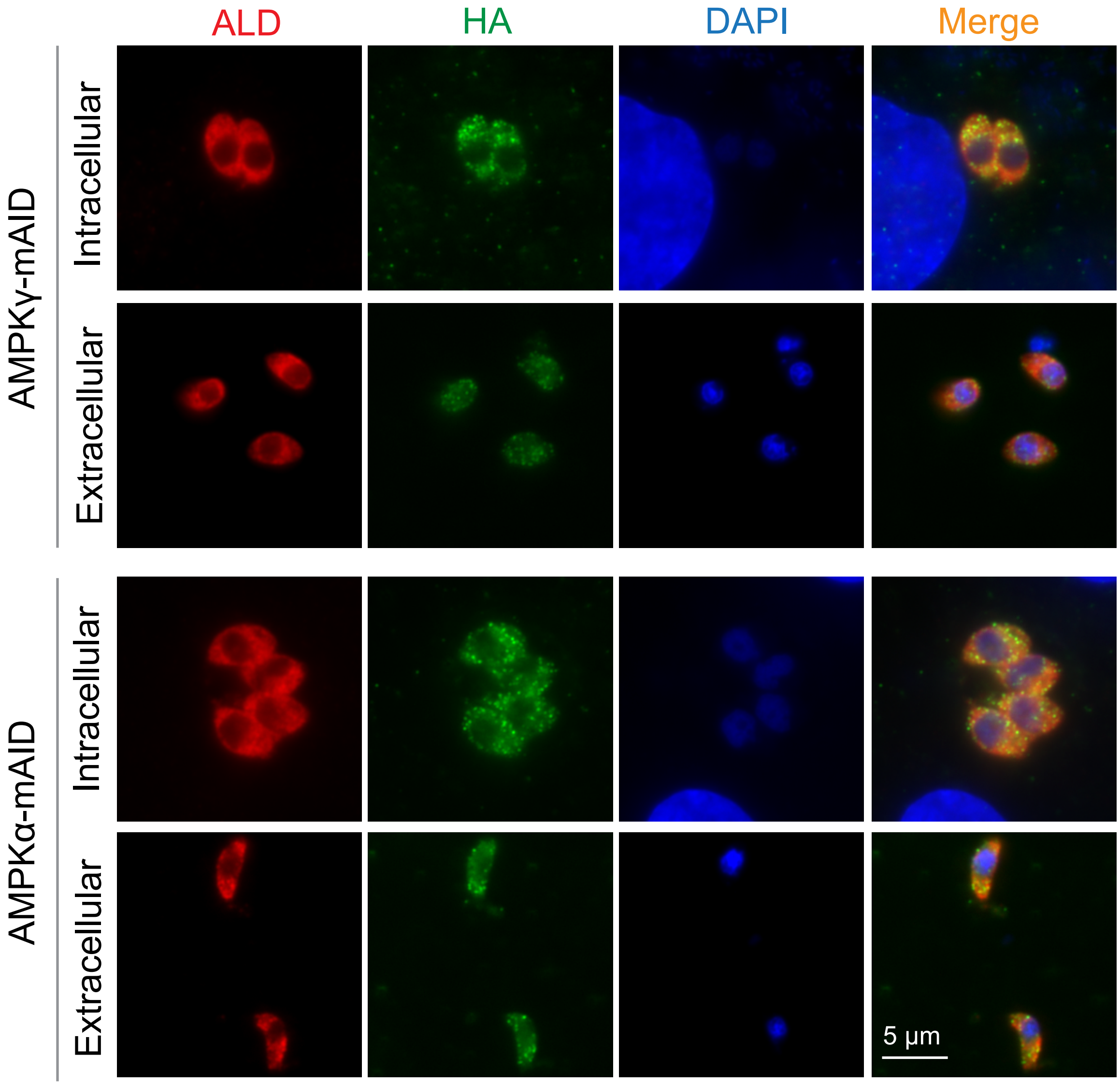

Fig S11. Localization of AMPKα and AMPKγ in intracellular and extracellular parasites of the AMPKα-mAID and AMPKγ-mAID strains (which contained an HA tag at the C-termini of endogenous AMPKα or AMPKγ). Intracellular or freshly released extracellular parasites obtained by syringe passage were fixed with paraformaldehyde, permeabilized with triton X-100 and probed with anti-ALD and anti-HA. AMPKα and AMPKγ were largely absent in parasite nuclei in intracellular parasites. However, more than 85% of AMPK γ-mAID and close to 50% of AMPKα-mAID parasites contained identifiable signal in the nuclei at the extracellular stage. Source data are provided as a Source data file.

Table S1. *Toxoplasma* proteins identified in all AMPK subunits based co-Ips

Gene ID	Product Description	AMPKα		AMPKβ		AMPKγ	
		Exp [1]	Control [2]	Exp [1]	Control [2]	Exp [1]	Control [2]
TGGT1_268960	AMPKβ	11	0	80	0	6	0
TGGT1_239870	AMPKγ	49	0	65	0	25	0
TGGT1_226910	Amylo-alpha-1,6-glucosidase	7	2	42	0	2	1
TGGT1_233905	AMPKα	49	0	28	0	14	0
TGGT1_253430	putative asparagine synthetase	10	4	18	9	10	5
TGGT1_289600	heat shock protein HSP29	2	1	14	0	2	1
TGGT1_278870	myosin F	16	4	12	1	1	0
TGGT1_286270	hypothetical protein	3	0	11	0	12	0
TGGT1_235020	putative COPI protein	12	1	8	0	1	0
TGGT1_263130	putative citrate synthase	7	1	8	1	2	1
TGGT1_268200	RNA recognition motif-containing protein	9	4	8	2	4	2
TGGT1_232130	hypothetical protein	8	4	7	3	4	2
TGGT1_266640	Acetyl-coenzyme A synthetase	11	2	7	0	6	3
TGGT1_228170	inner membrane complex protein IMC2A	15	1	6	2	2	1
TGGT1_263070	CMGC kinase, CK2 family	10	4	6	1	2	1
TGGT1_269980	putative preprotein translocase Sec61	10	5	6	3	5	2
TGGT1_292920	putative heat shock protein 75	9	2	6	2	1	0
TGGT1_313140	isocitrate dehydrogenase	8	2	6	2	2	1
TGGT1_213670	hypothetical protein	2	1	5	0	1	0
TGGT1_256770	putative eukaryotic translation initiation factor 4A, isoform 3	17	4	5	2	1	0
TGGT1_263870	glutamate-tRNA ligase	4	0	5	0	3	1
TGGT1_247470	putative nucleolar protein	11	4	4	2	1	0
TGGT1_289830	putative eukaryotic initiation factor-3, delta subunit	10	3	4	2	1	0

TGGT1_310640	phosphorylase family protein	1	0	4	1	3	1
TGGT1_229950	putative 26S proteasome regulatory subunit 6b	9	4	3	0	2	0
TGGT1_267080	putative 26S protease regulatory subunit 4	6	0	3	1	4	0
TGGT1_290660	RNA recognition motif-containing protein	7	0	3	1	6	3
TGGT1_310700	serine/threonine phosophatase PP1	8	2	3	0	2	0
TGGT1_312200	serine/threonine protein phosphatase	2	0	3	0	1	0
TGGT1_313230	eukaryotic initiation factor-2, alpha subunit	2	0	3	0	1	0
TGGT1_320020	transporter, major facilitator family protein	3	1	3	1	2	1
TGGT1_202390	S15 sporozoite-expressed protein	2	1	2	0	2	1
TGGT1_208560	carrier superfamily protein	1	0	2	0	1	0
TGGT1_209720	hypothetical protein	2	1	2	1	2	0
TGGT1_217820	PCI domain-containing protein	5	2	2	0	5	2
TGGT1_227960	PCI domain-containing protein	7	1	2	0	2	1
TGGT1_243800	putative long-chain fatty acid CoA ligase	3	1	2	0	2	0
TGGT1_243960	nuclear transport factor 2 (ntf2) domain-containing protein	4	0	2	0	2	0
TGGT1_245450	hypothetical protein	2	1	2	0	1	0
TGGT1_246580	hypothetical protein	5	1	2	1	2	0
TGGT1_283850	peptidyl-prolyl cis-trans isomerase	4	2	2	0	1	0
TGGT1_286630	redoxin domain-containing protein	2	1	2	0	1	0
TGGT1_295360	hypothetical protein	5	1	2	0	1	0
TGGT1_297810	hypothetical protein	4	2	2	0	5	2
TGGT1_202490	AP2 domain transcription factor AP2VIIa-7	2	1	1	0	1	0

TGGT1_202770	RNA recognition motif-containing protein	1	0	1	0	2	1
TGGT1_216590	HEAT repeat-containing protein	7	1	1	0	2	1
TGGT1_222380	importin-beta N-terminal domain-containing protein	2	0	1	0	1	0
TGGT1_223960	ubiquitin interaction motif family protein	2	0	1	0	1	0
TGGT1_226430	reticulon protein	2	1	1	0	2	1
TGGT1_233110	IMP dehydrogenase	3	0	1	0	1	0
TGGT1_237010	hypothetical protein	1	0	1	0	1	0
TGGT1_246940	hypothetical protein	1	0	1	0	1	0
TGGT1_247790	VRR-NUC domain-containing protein	1	0	1	0	1	0
TGGT1_255420	hypothetical protein	5	2	1	0	2	1
TGGT1_260820	IMC sub-compartment protein ISP1	6	3	1	0	6	3
TGGT1_261440	ARM repeats containing protein	2	0	1	0	1	0
TGGT1_262040	SAC3/GANP family protein	5	0	1	0	2	1
TGGT1_277500	putative 26S proteasome regulatory subunit 7	5	0	1	0	1	0
TGGT1_285980A	glucosephosphate-mutase GPM1	1	0	1	0	1	0
TGGT1_300060	signal peptidase subunit protein	2	1	1	0	2	1
TGGT1_309140	transducin beta-like protein TBL1	1	0	1	0	1	0
TGGT1_310150	AMP-binding enzyme domain-containing protein	5	0	1	0	3	0

[1] Number of unique peptides in the experimental group.

[2] Number of unique peptides in the corresponding control group.

Table S2. ***Toxoplasma gondii*** **strains used in this study**

Name	Description	Use	Source
RH Δ*hxgprt* Tir1	Express OsTir1, contain the *CAT* selection marker	For the construction of AID tagged strains	Reference 1
RH *Δhxgprt*	Type I strain RH with *HXGPRT* deletion	Parental strain	Dr. David Sibley
RH *Δhxgprt* /LoxP-*AMPKα-TY*	Express AMPKα-TY from the pTub promoter, contain the HXGPRT selection marker	For Co-IP to identify proteins interacting with AMPKα	This study
RH *Δhxgprt* /Loxp-*AMPKβ-TY*	Express AMPKβ-TY from the pTub promoter, contain the DHFR-Ts selection marker	For Co-IP to identify proteins interacting with AMPKβ	This study
AMPKγ-mAID	mAID-HA fused to the C terminus of endogenous *AMPKγ* in the RH Δ*hxgprt* Tir1 strain, contain the *HXGPRT* selection marker	For IAA induced depletion of AMPKγ in the RH Δ*hxgprt* Tir1 strain, as well as Co-IP to identify proteins interacting with AMPKγ	This study
Comp-γ	Expressing AMPKγ-TY by the pTub promoter from the *UPRT* locus of the AMPKγ-mAID strain, contain the DHFR-Ts selection marker	Complementation of the AMPKγ depletion strain AMPKγ-mAID	This study
AMPKγ-mAID /AMPKα-T221T-TY	Express AMPKα(T221T)-TY by the pTub promoter from the *UPRT* locus of the AMPKγ-mAID strain, contain the DHFR-Ts selection marker	To check the over-expression of AMPKα-T221T on the growth of the AMPKγ depletion strain AMPKγ-mAID	This study
AMPKγ-mAID /AMPKα-T221D-TY	Express AMPKα(T221D)-TY by the pTub promoter from the *UPRT* locus of the AMPKγ-mAID strain, contain the DHFR-Ts selection marker	To check the over-expression of AMPKα-T221D on the growth of the AMPKγ depletion strain AMPKγ-mAID	This study
AMPKγ-mAID /AMPKα-T221A-TY	Express AMPKα(T221A)-TY by the pTub promoter from the *UPRT* locus of the AMPKγ-mAID strain, contain the DHFR-Ts selection marker	To check the over-expression of AMPKα-T221A on the growth of the AMPKγ depletion strain AMPKγ-mAID	This study
AMPKα-mAID	mAID-HA fused to the C terminus of endogenous *AMPKα* in the RH Δ*hxgprt* Tir1 strain, contain the *HXGPRT* selection marker	For IAA induced depletion of AMPKα in the RH Δ*hxgprt* Tir1 strain	This study

Table S3. Plasmids used in this study

Plasmids	**Construction methods**	**Use**
pSAG1-Cas9-sgUPRT	Reference 2	*UPRT*-specific CRISPR plasmid and template for gene-specific CRISPR plasmid construction
pSAG1::Cas9-sgAMPKγ-3UTR	Replace sgUPRT in pSAG1-Cas9-sgUPRT with sgAMPKγ	*AMPKγ* specific CRISPR plasmid, for the construction of AMPKγ-mAID strain
pTUB1:YFP-mAID-3HA- HXGPRT	Reference 1	Template for mini-AID-HXGPRT amplification
pUC19-AMPKγ-3HA-HXGPRT	Clone mini-AID-HXGPRT, 5' and 3' homology arms of AMPKγ into pUC19	Homology template for the construction of AMPKγ-mAID
pTUB1-GOI-Ty-YFP-DHFR*	GOI-Ty-YFP fusion driven by the TUB1 promoter with the DHFR* as drug selectable marker.	Template for pG265-UPRT::AMPKγ-DHFR* construction
pSAG1:Cas9-sgAMPKα	Replace sgUPRT in pSAG1-Cas9-sgUPRT with sgAMPK*α*	AMPKα specific CRISPR plasmid, for the construction of RH *Δhxgprt* /LoxP-AMPKα-TY strain
pDONR-G265	From the Sibley Lab	Template for pTUB1: LoxP- killer-red -LoxP-YFP- HXGPRT amplification
pTUB1::AMPKα-Ty-YFP- DHFR*	Clone the AMPKα coding sequence into pTUB1-GOI-Ty-YFP-DHFR*	Intermediate plasmid for pTUB1::5H-AMPK-Ty-YFP- DHFR* -3H
pTUB1::5H-AMPK-Ty-YFP- DHFR* -3H	Insert the 5' and 3' homology arms of AMPKα into pTUB1::AMPKα-Ty-YFP- DHFR*	Homology template for the construction of RHΔhxgprt /LoxP-AMPKα-TY strain
pSAG1:Cas9-sgAMPKβ	Replace sgUPRT in pSAG1-Cas9-sgUPRT with sg AMPKβ	AMPKβ specific CRISPR plasmid, for the construction of RH Δhxgprt /LoxP-AMPKβ-TY strain
pG265- 5H-LoxP-killer-red-LoxP-YFP-HXGPRT-3H	Insert the 5' and 3' homology arms of AMPKβ into pDONR-G265	Intermediate plasmid for pTUB1-Loxp-AMPKβ-TY-LoxP-YFP-HXGPRT
pTUB1-Loxp-AMPKβ-TY-LoxP-YFP-HXGPRT	Replace killer-red in pDONR-G265 with AMPKβ-TY	Homology template for the construction of RH Δhxgprt /Loxp-AMPKβ-TY strain
pG265-UPRT::AMPKγ-DHFR*	Clone the AMPKγ coding sequence into pTUB1-GOI-Ty-YFP-DHFR*	Deliver the *AMPKγ* Expressing cassette to the *UPRT* locus, for the construction of the complementation strain Comp-γ. It was also used as template for the construction of pG265-UPRT::AMPKα-T221T/A/D-DHFR*
pG265-UPRT::AMPKα 221T- DHFR *	Replace AMPKγ in pG265-UPRT::AMPKγ-DHFR* with the coding sequence of AMPKα	Homology template for the construction of AMPKγ-mAID /AMPKα-T221T-TY strain
pG265-UPRT::AMPKα 221D- DHFR*	Replace AMPKγ in pG265-UPRT::AMPKγ-DHFR* with the coding sequence of AMPKα-221D	Homology template for the construction of AMPKγ-mAID /AMPKα-T221D-TY strain
pG265-UPRT::AMPKα 221A- DHFR*	Replace AMPKγ in pG265-UPRT::AMPKγ-DHFR* with the coding sequence of AMPKα-221A	Homology template for the construction of AMPKγ-mAID /AMPKα-T221A-TY strain
pSAG1:Cas9-sgAMPKα(AID)	Replace sgUPRT in pSAG1-Cas9-sgUPRT with sg AMPKα(AID)	AMPKα specific CRISPR plasmid, for the construction of AMPKα-mAID strain
pAMPKα-mAID	Clone mini-AID-HXGPRT, 5' and 3' homology arms of AMPKα into pUC19	Homology template for the construction of AMPKα-mAID strain

Table S4. Primers used in this study

Primers	Sequence (5'-3')	Use
AMPKγ-AID-sgRNA-F	GGAACGGCCAGACGTCGCCTGTTTTAGAGCTAGAAATAGC	To construct the *AMPKγ*-specific CRISPR plasmid pSAG1::Cas9-U6::sg-AMPKγ-3UTR
sgRNA-R	AACTTGACATCCCCATTTAC	
pUC19-AID-F	GGTACCCGGGGATCCTCTAGA	Amplify the pUC19 backbone from pTUB1:YFP-mAID-3HA-HXGPRT for pUC19-AMPKγ-3HA-HXGPRT construction
pUC19-AID-R	CATATGGTGCACTCTCAGTACAATCTG	
AMPKγ-AID-F	CCTAGGATGGTGAGCGCTAGCA	Amplify mini-AID-*HXGPRT* from pTUB1:YFP-mAID-3HA-HXGPRT for pUC19-AMPKγ-3HA-HXGPRT construction
AMPKγ-AID-R	CCCATTCGCCATTCAGGCTG	
AMPKγ-AID-5H-F	TACTGAGAGTGCACCATATG ACAGAAGTCCAGCGGATCTGGG	Amplify the 5' homologous arm from gDNA of *AMPKγ* for pUC19-AMPKγ-3HA-HXGPRT construction
AMPKγ-AID-5H-R	TGCTAGCGCTCACCATCCTAGGATCTGAGGAAATCCTCTGG	
AMPKγ-AID-3H-F	CAGCCTGAATGGCGAATGGGGACTGACTCACTCTGGAAAG	Amplify the 3' homologous arm from gDNA of *AMPKγ* for pUC19-AMPKγ-3HA-HXGPRT construction
AMPKγ-AID-3H-R	CTAGAGGATCCCCGGGTACCCGGCCGTACGTCAGAAAATAG	
AID-PCR1-F	CATTTCTATGCGGCCCAGAG	Diagnostic PCRs for the identification of AMPKγ-mAID clones
AID-PCR1-R	GGCAAGAGACCATCACGTTC	
AID-PCR2-F	GCGTTGGCCTACGTGACTTG	
AID-PCR2-R	AACACAGAGCGCCGTTCAG	
AID-PCR3-F	GAAGGCCAAGAAGGCGAAAG	
AID-PCR3-R	CTCTGCGCTGCATTTCTGTC	
pDG265-F	GAGGTCCACACGAACCAGGA	Amplify the vector backbone from pTUB1-GOI-Ty-YFP-DHFR* for pG265-UPRT::AMPKγ-DHFR* or pG265-UPRT::AMPKα 221T-DHFR*construction
pDG265-R	TTTGTCGGAATTCTATAACTTCGTATAA	
ampkγ-CDS-F	GAAGTTATAGAATTCCGACAAAATGTCGCGCAGAGAAGAAGT	Amplify the *AMPKγ* coding sequence from cDNA of RH for pG265-ptublin-UPRT:: AMPKγ-DHFR* construction
ampkγ-CDS-R	GTCCATGCCGAGAGTGATCCTTAATCGAGCGGGTCCTGGT	
AMPKγ-comp-F	GGCTAGGCGATTAAGTTGGG	Amplify the UPRT:: AMPKγ-DHFR* fragment from pG265-
AMPKγ-comp-R	GATTCCGTCAGCGGTCTGTC	

		UPRT::AMPKγ-DHFR* to construct the Comp-γ strain.
comp-PCR4-F	CTCCTTGGACGTGGACGTTTC	Diagnostic PCRs for the identification of Comp-γ clones.
comp-PCR4-R	TCCTGGTTCGTGTGGACCTC	
comp-PCR5-F	CATGACCCACTTCAGTCTAC	
comp-PCR5-R	CCTCTTGCCTCCATAGTTTC	
T221T-F	AGTTATAGAATTCCGACAAAATGTGC ACGCCGGCATGG	Amplify *AMPKα*-221T from cNDA for the construction of pG265-ptublin-UPRT::AMPKα-221T-DHFR*
T221T-R	TCCTGGTTCGTGTGGACCTCCAGCCC CCCGGTATCTATTC	
T221D-F	GAGATGGAGACTTTTTGAAAGACTCT TGTGGGTCTCCGAA	Amplification of AMPKα T221D-Vector for pG265-ptublin-UPRT::AMPKα T221A-DHFR*
T221D-R	TTCGGAGACCCACAAGAGTCTTTCA AAAAGTCTCCATCTC	
T221A-R	TTCGGAGACCCACAAGATGCTTTCAA AAAGTCTCCATCTC	Amplification of *AMPKα* T221A-Vector for pG265-ptublin-UPRT::AMPKα T221A- DHFR*
T221A-F	GAGATGGAGACTTTTTGAAAGCATCT TGTGGGTCTCCGAA	
AMPKα-PCR4-F	CCCGAACATGAAGCGAGGAG	Diagnostic PCRs for the identification of AMPKγ-mAID/AMPKα-221T/A/D clones.
comp-PCR4-R	TCCTGGTTCGTGTGGACCTC	
comp-PCR5-F	CATGACCCACTTCAGTCTAC	
comp-PCR5-R	CCTCTTGCCTCCATAGTTTC	
gRNA-AMPKβ-F	ACCTCAGACCGGTACCAGTG GTTTTAGAGCTAGAAATAGC	To construct the *AMPKβ* specific CRISPR plasmid pSAG1:Cas9-U6: sgAMPKβ
5H-AMPKβ-F	CGTACCGCTAGCCAGGAAGAATGCTG GCGGTGCTTGTTC	Amplify the 5’-homology of AMPKβ from gDNA for pDONR-G265-5H-TUB1: loxP- killer-red -loxP-YFP- HXGPRT -3H
5H-AMPKβ-R	GAGCTTAAGACTGGCCGTCGCATGAC GCTCTGAAGCTCG	
TUB1-Loxp-killer-red-Loxp-YFP -HXGPRT-F	CGACGGCCAGTCTTAAGCTC	Amplify the TUB1-Loxp-killer-red-Loxp-YFP -HXGPRT fragment from pDONR-G265 for pDONR-G265-5H-
TUB1-Loxp-killer-red-Loxp-YFP -HXGPRT-R	CGCGCAATTAACCCTCACTA	

		TUB1: loxP- killer-red -loxP-YFP- HXGPRT -3H
3H-AMPKβ-F	TAGTGAGGGTTAATTGCGCGCAGATG CTACGTGGCTATCC	Amplify the 3'- homology of AMPKβ from gDNA for pDONR-G265-5H-TUB1: loxP- killer-red -loxP-YFP- HXGPRT -3H
3H-AMPKβ-R	GCTATGACCATGATTACGCCTATGTCG TGTCTCCAGCGTG	
pDONR-G265-vector- AMPKβ-F	GGCGTAATCATGGTCATAGC	Amplify the Amplification of the vector backbone from pDONR-G265 for pDONR-G265-5H-TUB1: loxP- killer-red -loxP-YFP- HXGPRT -3H
pDONR-G265-vector- AMPKβ-R	TCTTCCTGGCTAGCGGTACG	
AMPKβ-CDS-F	ATAGAATTCCGACAAAATGGGATCTC AGACGAGCAACAGT	Amplify the AMPKβ-CDS-Ty fragment from cDNA for pDONR-G265-5H-TUB1: LoxP-AMPKβ-TY-LoxP-YFP-HXGPRT-3H
AMPKβ-CDS-Ty-R	TTAATCGAGCGGGTCCTGGTTCGTGT GGACCTCGGAAGACACACTTGGCGT CTC	
AMPKβ-CDS-vector-F	ACCAGGACCCGCTCGATTAACGAGG ATATGCATAGATCTT	Amplify the vector backbone of AMPKβ-CDS-Ty fragment for pDONR-G265-5H-Tub: LoxP-AMPKβ-TY-LoxP-YFP- HXGPRT -3H construction
AMPKβ-CDS-vector-R	CCATTTTGTCGGAATTCTAT	
AMPKβ-PCR1-F	TCTATCGAACTGCCCACTCC	Diagnostic PCRs for the identification of RH *Δhxgprt* /Loxp-AMPKβ-TY clones
AMPKβ-PCR1-R	CAACTTAATCGCCTAGCCTG	
AMPKβ-PCR2-F	GCGGTGGAGCTCTGATCAGG	
AMPKβ-PCR2-R	GATGGCATGGTGACACTTAG	
AMPKβ-PCR3-F	GGATCTCAGACGAGCAACAG	
AMPKβ-PCR3-R	TGAAGACGCATGGCGTGAG	
F-AMPKα CDS	agttatagaattccgacaaaATGTGCACGCCGG CATGG	Amplify the AMPKα coding sequence from cDNA of RH for
R-AMPKα CDS	tcctggttcgtgtggacctcCAGCCCCCCGGTAT CTATTC	

		pTUB1::AMPKα-Ty-YFP- DHFR*
pDG265-F	GAGGTCCACACGAACCAGGA	Amplify the vector backbone from pTUB1-GOI-Ty-YFP-DHFR* for pTUB1::AMPKα-Ty-YFP- DHFR*
pDG265-R	TTTGTCGGAATTCTATAACTTCGTATAA	
F-5H- AMPKα	attgtactgagagtgcaccaACCCCAAAAAGGATTCGCG	Amplify the 5' homologous arm from gDNA of AMPKα for pTUB1::5H-AMPK-Ty-YFP- DHFR* -3H
R-5H- AMPKα	taagactggccgtcgTGTCTCCGGAACGAGAGTCG	
F-AMPKα-Ty-YFP-DHFR*	agacaCGACGGCCAGTCTTAAGCTC	Amplify the AMPKα-Ty-YFP-DHFR* from pTUB1::AMPKα-Ty-YFP- DHFR* fragment for pTUB1::5H-AMPK-Ty-YFP- DHFR* -3H
R-AMPKα-Ty-YFP-DHFR*	tttttgccGATTCCGTCAGCGGTCTGTC	
F-3H- AMPKα	gctgacggaatcGGCAAAAAAGAGAGTGTAGAACGA	Amplify the 3' homologous arm from gDNA of AMPKα for pTUB1::5H-AMPK-Ty-YFP- DHFR* -3H
R-3H- AMPKα	cgactctagaggatccccggGCTATGTACGTACACACG	
F-vector- AMPKα	CCGGGGATCCTCTAGAGTCG	Amplify the vector backbone from pTUB1::AMPKα-Ty-YFP- DHFR* for pTUB1::5H-AMPK-Ty-YFP- DHFR* -3H
R-vector- AMPKα	TGGTGCACTCTCAGTACAATCTGC	
F-PCR1-AMPKα	TTCGCCGGACAAAAGAAGAG	Diagnostic PCRs for the identification of RH *Δhxgprt* /LoxP-AMPKα-Ty clones
R-PCR1-AMPKα	TTTGTCGGAATTCTATAACTTCGTATAA	
F-PCR2-AMPKα	GAGGTCGTGGGCTACGTCCC	
R-PCR2-AMPKα	GCTGAAGCGGTGATACGGCG	
F-PCR3-AMPKα	GAAAACCTCTTGTGGGTCTC	
R-PCR3-AMPKα	TCATCTGCAAGACGATGAG	

Supplemental references

1. Brown KM, Long S, & Sibley LD (2017) Plasma Membrane Association by N-Acylation Governs PKG Function in Toxoplasma gondii. *mBio* 8(3).
2. Shen B, Brown KM, Lee TD, & Sibley LD (2014) Efficient gene disruption in diverse strains of Toxoplasma gondii using CRISPR/CAS9. *mBio* 5(3):e01114-01114.

Unprocessed blots and uncropped gels for supplementary figures

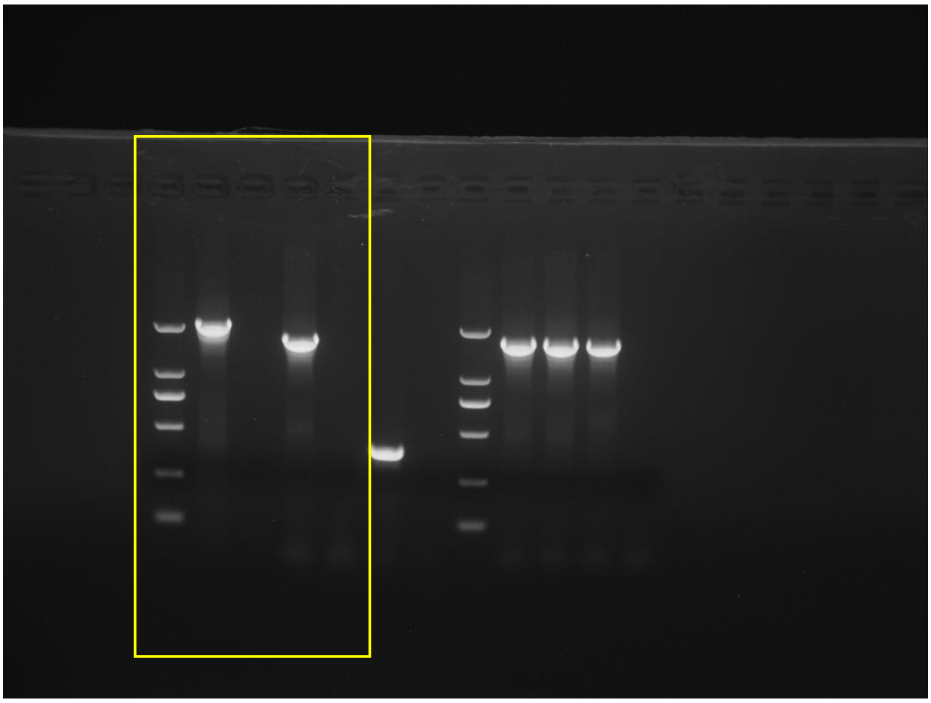

Fig S1b. PCR1/PCR2.

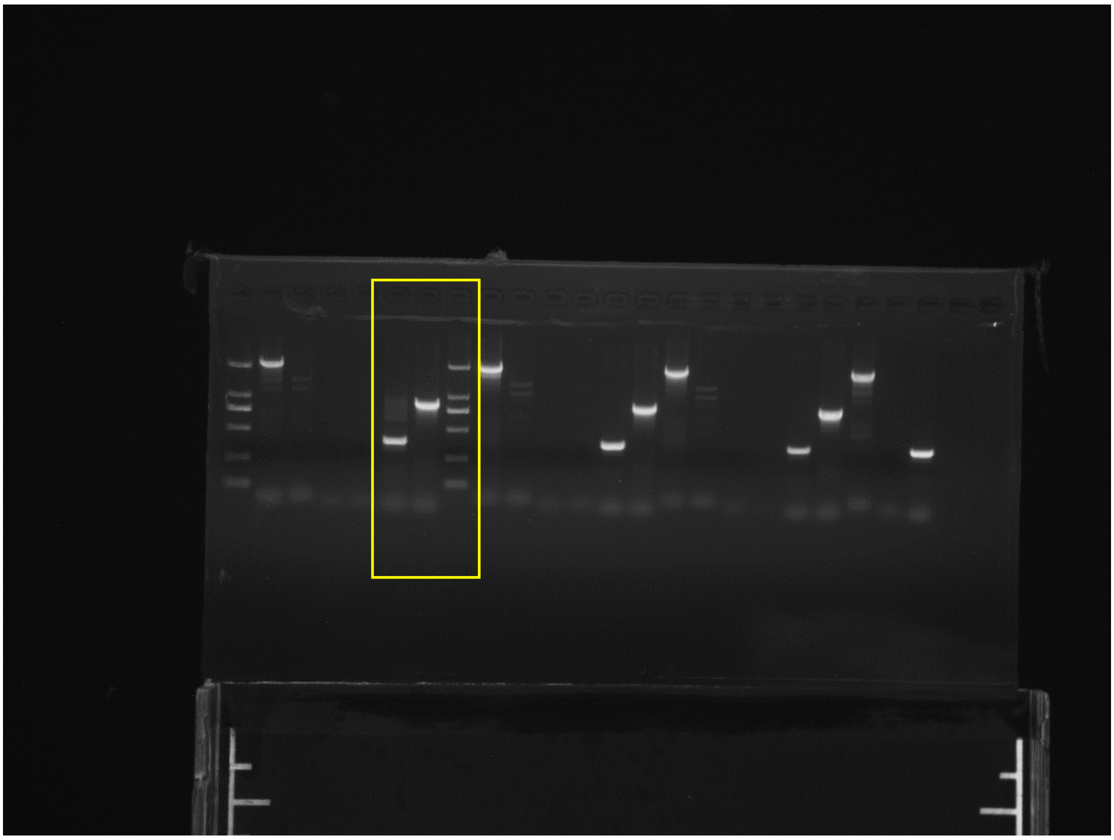

Fig S1b. PCR3.

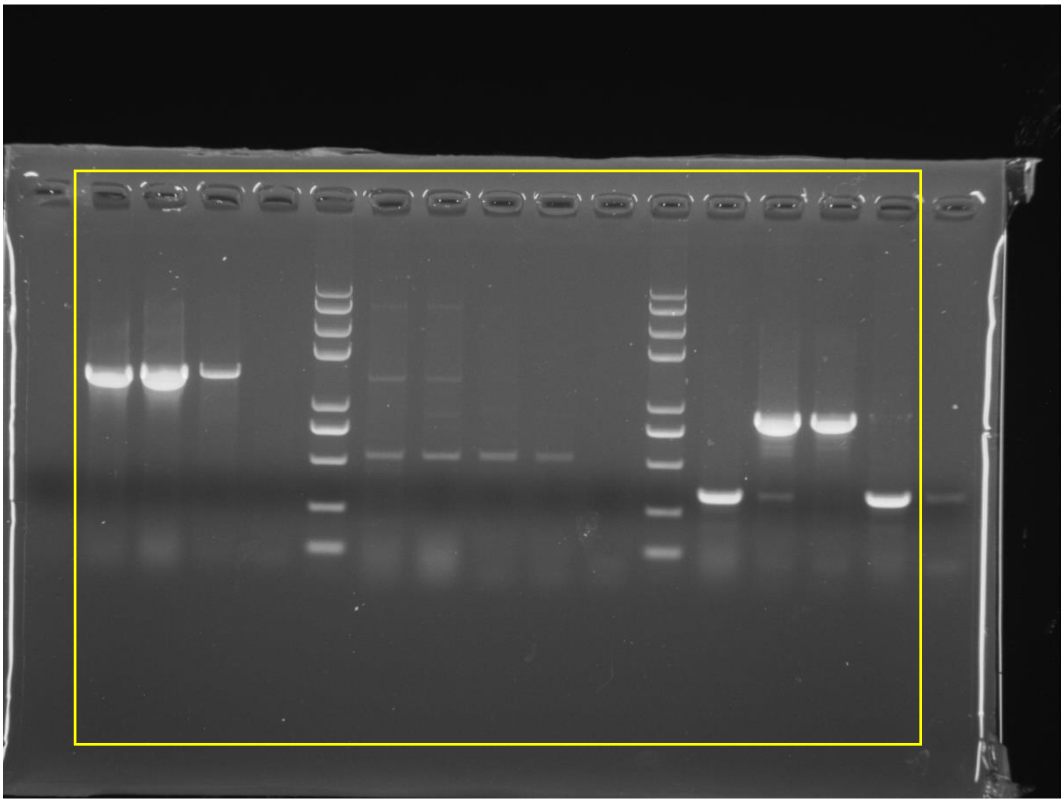

Fig S2b.

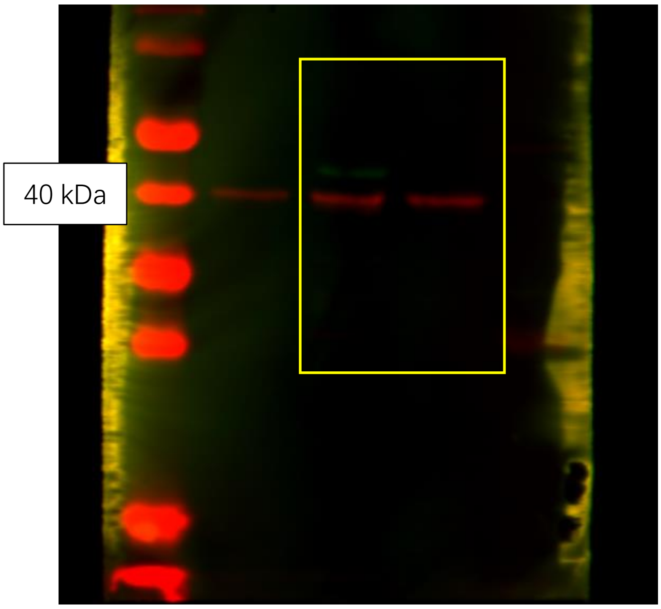

Fig S2c.

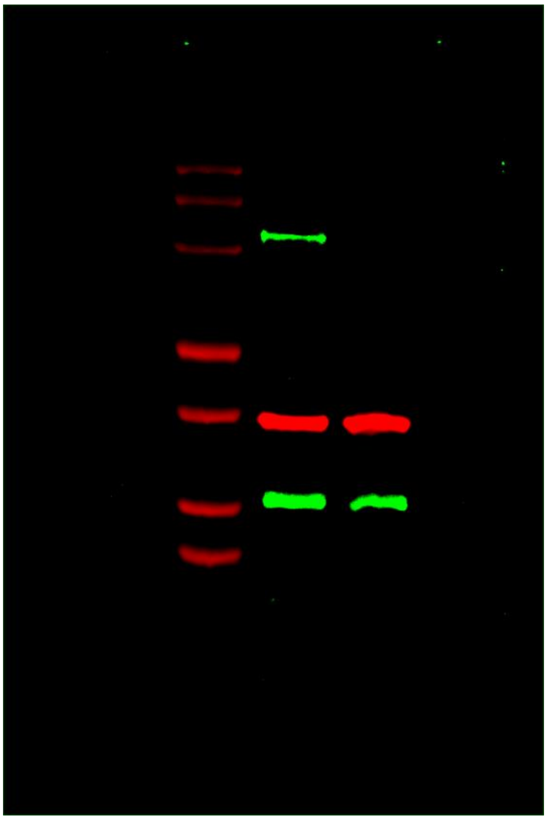

Fig S4a.

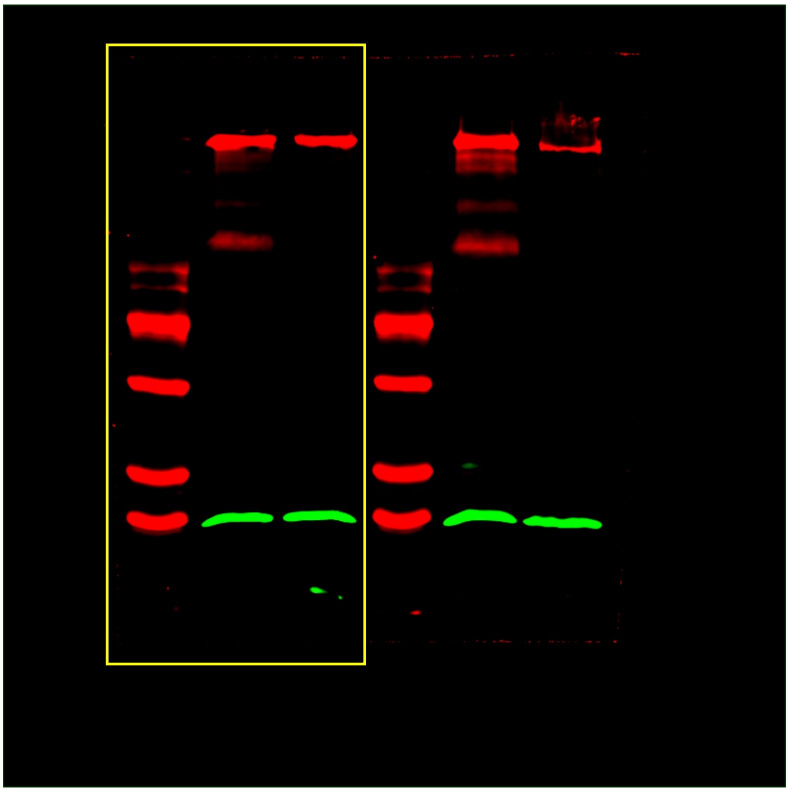

Fig S4b.

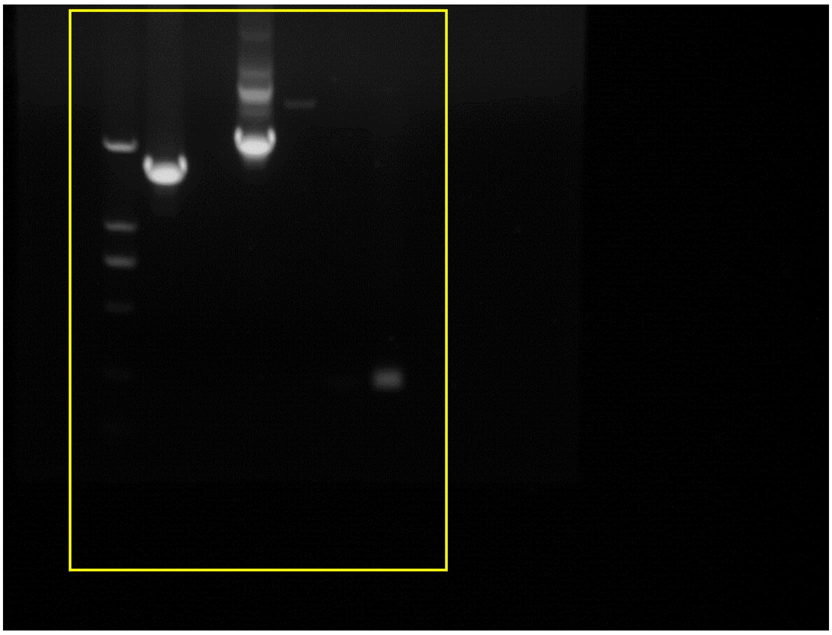

Fig S5b.

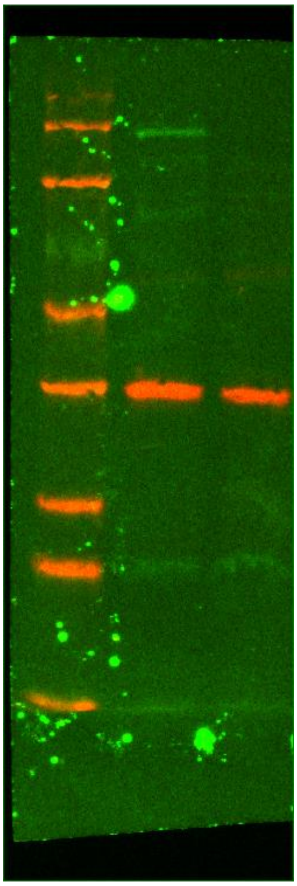

Fig S5c

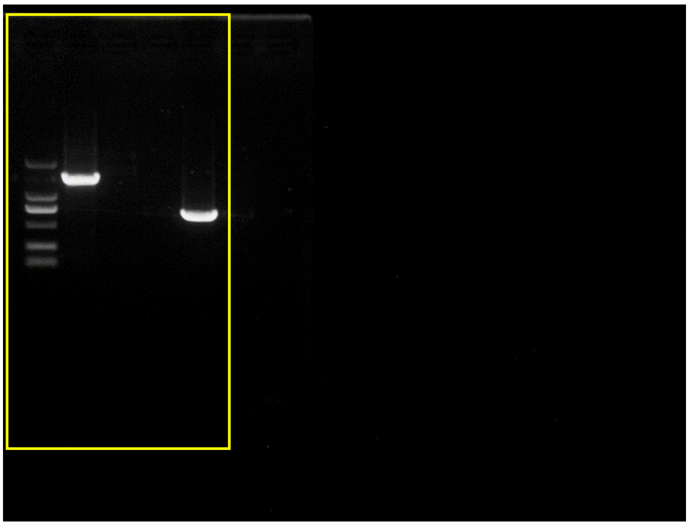

Fig S6b

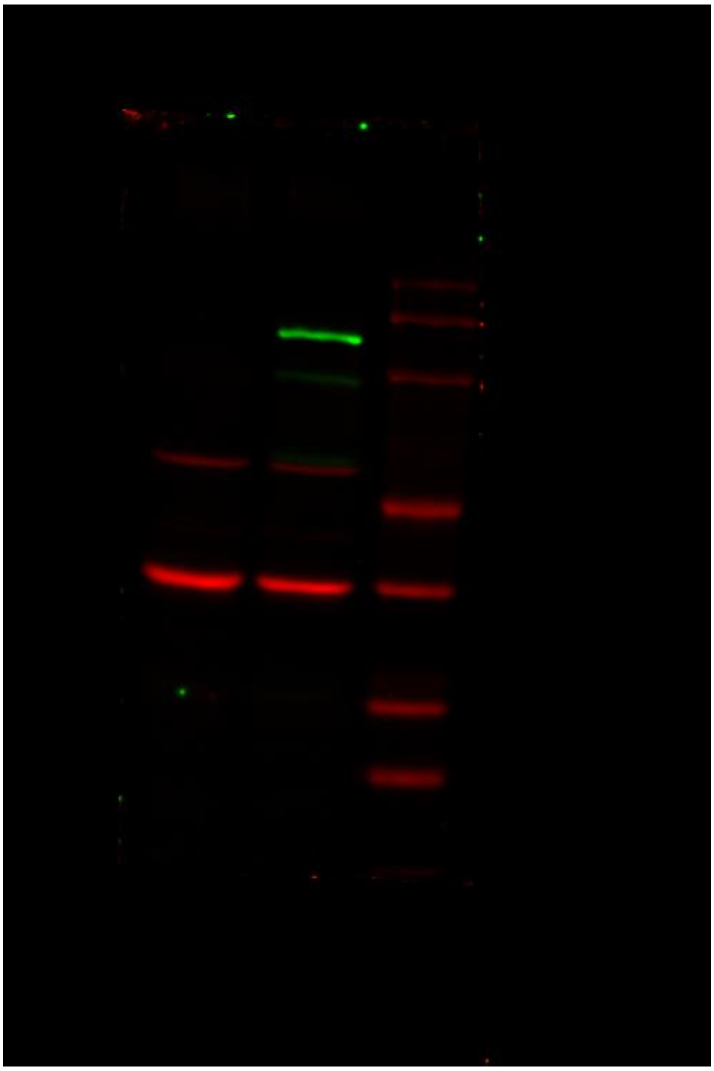

Fig S6c

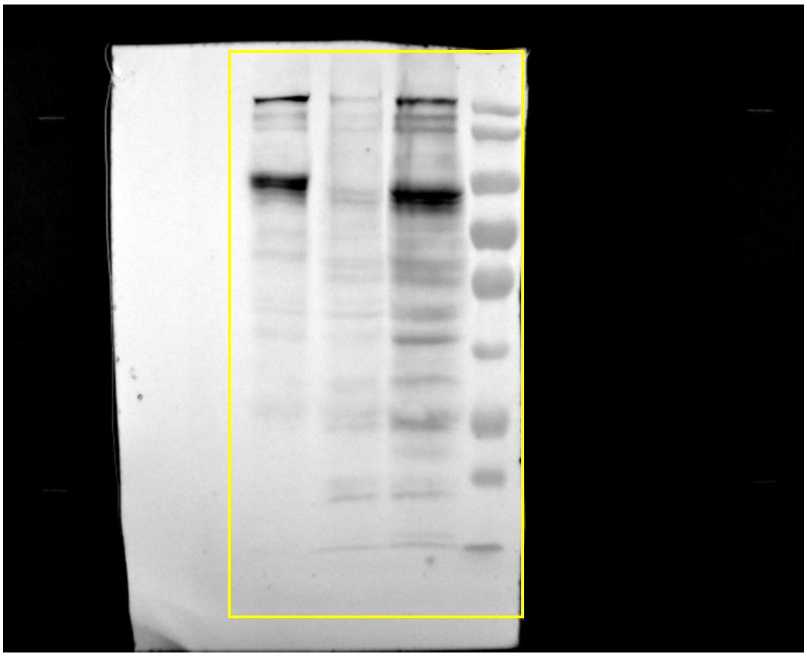

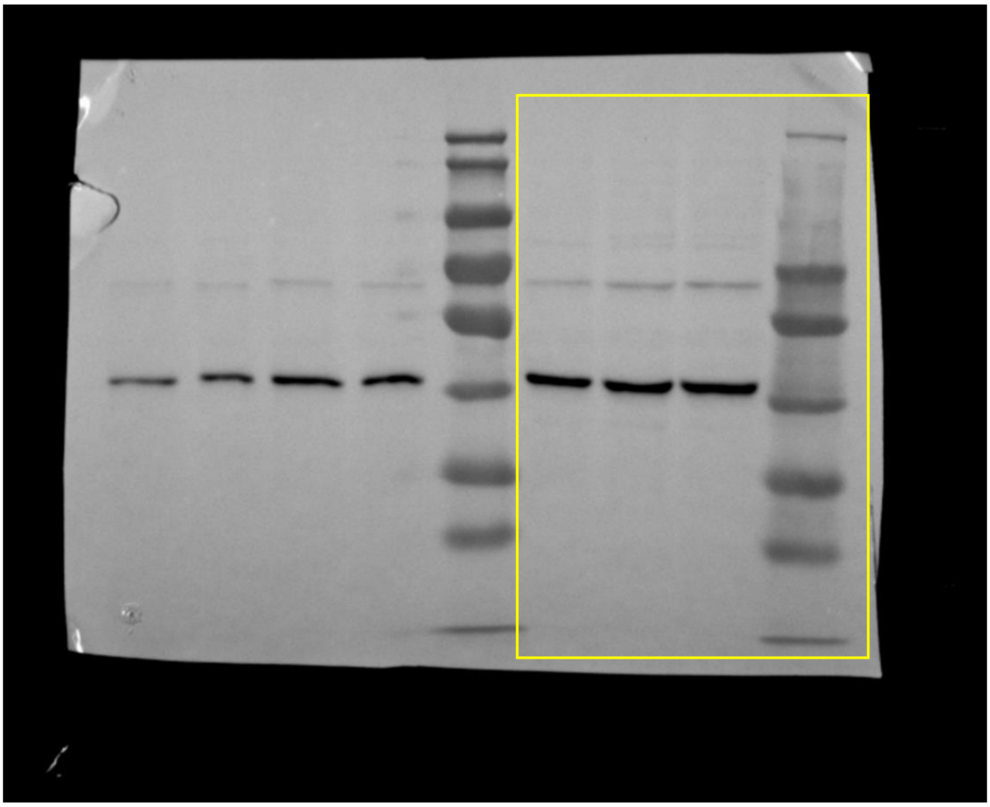

Fig S7
