# Supplementary material for: Rapid metabolic reprogramming mediated by the AMP-activated protein kinase during the lytic cycle of Toxoplasma gondii
Source: Nat Commun. 2023 Jan 26;14:422. doi: 10.1038/s41467-023-36084-0 (PMC9880002; doi:10.1038/s41467-023-36084-0)
Supplement: Supplementary file 2 — Description of Additional Supplementary Files [file 41467_2023_36084_MOESM2_ESM.docx]

**Description of Additional Supplementary Files**

**File Name: Supplementary data 1.** Toxoplasma proteins co-precipitated with each of the AMPK subunit.

**File Name: Supplementary data 2.** Differentially expressed genes after AMPKγ depletion revealed by RNA-Seq.

**File Name: Supplementary data 3.** Proteins with significant abundance change upon AMPKγ depletion.

**File Name: Supplementary data 4.** Toxoplasma phosphopeptides and their abundance changes in the AMPKγ-mAID strain treated with and without IAA.
